# Arginyltransferase1 drives a mitochondria-dependent program to induce cell death

**DOI:** 10.1038/s41419-025-07917-1

**Published:** 2025-08-16

**Authors:** Akhilesh Kumar, Corin R. O’Shea, Vikas K. Yadav, Ganapathi Kandasamy, Balaji T. Moorthy, Evan A. Ambrose, Aliya Mulati, Flavia Fontanesi, Fangliang Zhang

**Affiliations:** 1 Department of Molecular & Cellular Pharmacology, University of Miami Miller School of Medicine, Miami, FL USA; 2 Graduate Program of Molecular & Cellular Pharmacology, University of Miami Miller School of Medicine, Miami, FL USA; 3 Department of Botany, Banaras Hindu University, Varanasi, India; 4 Graduate Program of Cancer Biology, University of Miami Miller School of Medicine, Miami, FL USA; 5 Department of Biochemistry & Molecular Biology, University of Miami Miller School of Medicine, Miami, FL USA; 6 Sylvester Comprehensive Cancer Center, University of Miami Health System, Miami, FL USA; 7 Present Address: Department of Botany, Banaras Hindu University, Varanasi, India; 8 Present Address: Sanofi, Waltham, MA USA

**Keywords:** Cell death, Post-translational modifications

## Abstract

Cell death regulation is essential for stress adaptation and/or signal response. Past studies have shown that eukaryotic cell death is mediated by an evolutionarily conserved enzyme, arginyltransferase1 (Ate1). The downregulation of Ate1, as seen in many types of cancer, prominently increases cellular tolerance to a variety of stress conditions. Conversely, in yeast and mammalian cells, Ate1 is elevated under acute oxidative stress conditions, and this change appears to be essential for triggering cell death. However, studies of Ate1 were conventionally focused on its function in inducing protein degradation via the N-end rule pathway in the cytosol, leading to an incomplete understanding of the role of Ate1 in cell death. Our recent investigation shows that Ate1 dually exists in the cytosol and mitochondria, the latter of which has an established role in cell death initiation. Here, by using budding yeast as a model organism, we found that mitochondrial translocation of Ate1 is promoted by the presence of oxidative stressors, and this process is essential for inducing cell death preferentially through the apoptotic pathway. Also, we found that Ate1-induced cell death is dependent on the formation of the mitochondrial permeability transition pore and at least partly dependent on the action of mitochondria-contained factors, including the apoptosis-inducing factor, but is not directly dependent on mitochondrial electron transport chain activity or reactive oxygen species (ROS) derived from it. Furthermore, our evidence suggests that, contrary to widespread assumptions, the cytosolic protein degradation pathways, including ubiquitin-proteasome, autophagy, or endoplasmic reticulum (ER) stress response, has little or negligible impacts on Ate1-induced cell death in the tested conditions. We conclude that Ate1 controls the mitochondria-dependent cell death pathway.

## Introduction

The regulation of programmed cell death (PCD) in eukaryotes is essential for the organism to properly respond to stress conditions, to restrict the temporal impact of damages, to adapt to chronic stress, and to shape the tissues during development and regeneration. As such, the understanding of PCD events possesses the utmost significance for understanding eukaryotic life forms. In human, dysregulation of PCD is directly responsible for many types of diseases or abnormalities such as cancer, diabetes, autoimmunity, aging-related disorders, prenatal deformation, and embryonic lethality.

Studies from us and other groups showed that eukaryotic PCD is influenced by arginyltransferase (*ATE*) family proteins, which is known for being the only enzyme family mediating arginylation in eukaryotes. Arginylation is the posttranslational addition of an extra arginine to target proteins independently of the ribosome and mRNA [1]. This modification may take place on an eligible N-terminus or side chain of substrate proteins. By adding a charged and bulky chemical group, arginylation is expected to lead to significant changes of the properties of the substrate protein. When taking place at the N-terminus, arginylation often leads to hyper-ubiquitination and/or rapid degradation of the target protein by the proteasome or autophagy system via the N-end rule pathway [2, 3]. In all eukaryotes, the *ATE* genes are coded in the nuclear genome. While in plants the *ATE* family include two isoforms (Ate1 and Ate2) due to gene duplication, most other eukaryotes (such as fungi, animals, and humans) only carry one isoform, Ate1.

The protein level and/or the activity of Ate1 protein were found to increase upon the acute exposure to a broad range of stressors, including oxidants, heavy metal, physical injury, and radiations [4–9]. Conversely, the decreased expression or inhibition of Ate1 often renders the cells insensitive to these stress factors [9–11]. These data suggested that Ate1 may have a direct role in regulating cell death. Indeed, our past study showed that the ectopic overexpression of Ate1, even in the absence of exogenous stressors, is a potent inducer of cell death [9]. Consistently, multiple genetic studies uncovered many Ate1-associated phenotypes in embryo/tissue development and/or diseases that can be attributed to PCD regulation. For example, the knockout (KO) of Ate1 gene (*ATE1*) in animals leads to complete embryonic lethality at the early-mid gestation stage [12]. Conditional deletion or dysregulation of *ATE1* also causes problems in cardiovascular development, neuronal development, and angiogenesis [13–16]. Furthermore, downregulation of Ate1 appears to contribute to the development and progression of cancer, which is often coupled with decreased PCD [11, 17].

In the attempts of addressing the role of Ate1/arginylation in PCD, two approaches have been used in past studies. In the first one, genetic manipulations or specific inhibitor were used to study the global consequences of interfering with the level/activity of Ate1 and its functional partners. In most cases, the suppression of Ate1 or its functional partners N-terminal amidohydrolase or ubiquitin-protein ligase E3 component N-recognin (UBR), drastically decreases cellular sensitivities to death-inducing stressors [10, 18–20], while direct upregulations of these corresponding elements often promote cell death [9]. As such, most of these data implied an overall pro-apoptotic role for Ate1/arginylation. In the second approach, researchers focused on identifying arginylation substrate proteins in the cytosol that are involved in PCD-related processes. These processes include protein degradation and/or quality-check machineries such as the ubiquitin-proteasome system (UPS), autophagy, and unfolded protein response (UPR) in the endoplasmic reticulum (ER) [21–23]. The identified Ate1 substrates include full-length proteins or proteolytic fragments generated by caspase or calpain. Many of the identified proteins/fragments appear to have known apoptosis-promoting roles (if present in the cytosol). Examples include BRAC1, RIPK1, and EPHA4. Since Ate1-mediated arginylation appears to accelerate their turnovers, this naturally led to the hypothesis of an anti-apoptotic role of Ate1/arginylation [24–27]. However, this speculation is at odds with the result from the first approach mentioned above. It is also at odds with other target-based studies, including finding showing that arginylation of certain autophagy components (such as the cytosolic BiP) promotes autophagy and reduce PCD [22, 28]. These discrepancies indicated that the role of Ate1/arginylation in PCD is likely more complex than previously thought and that there is likely a major missing mechanistic link between the different approaches.

A new insight for the investigation of Ate1 was provided in our recent study showing a previously overlooked connection between Ate1 and mitochondria, the latter of which is a major regulator of PCD. In general, cell death can be triggered by the presence of stressors such as reactive oxygen species (ROS) or DNA damages, or by the signalling relayed by receptors for extracellular ligands (such as cytokines). These stimuli further activate intracellular signalling/actioning proteins such as the caspases to initiate the PCD process. A major regulator of PCD is mitochondria, which are the main generators of bioenergetics molecules (ATP) as well as endogenous ROS as a byproduct of electron transport chain (ETC) activity. Furthermore, a variety of stressing conditions or signalling events can cause mitochondrial membrane leakage, leading to the release of otherwise mitochondria-contained pro-death factors such as cytochrome *c* (Cyc), apoptosis-inducing factor (Aif), and endonuclease G (Nuc1). The release of these factors to the cytosol often triggers cell death events in caspase-dependent and independent manners [29, 30]. Our analysis revealed that the eukaryotic *ATE1* gene, which is hosted in the nuclear genome, was likely derived from mitochondrial gene transfer during early eukaryote evolution. We also found that, while the absolute majority of Ate1 resides in the cytosol and nucleus, a small fraction of this protein is located inside mitochondria [31]. Consistently, studies from us and others showed that Ate1 is essential for maintaining normal mitochondrial respiration and/or morphology in mammalian and yeast cells [31, 32]. Considering the known role of mitochondria as a central hub for initiating and coordinating PCD, we were inspired to question whether Ate1 regulates PCD through mitochondria-dependent pathways. However, past studies about Ate1 were nearly exclusively examining its actions in the cytosol or nucleus, lending little clue to address the role of Ate1 in relation to mitochondria.

To test how Ate1 regulates cell death, in this study, we took advantage of the budding yeast (*Saccharomyces cerevisiae*) as a test model, for which well-established assays for PCD and Ate1/arginylation are readily available. In this organism, PCD is mediated by an ensemble of machineries that are conserved among other eukaryotic organisms. As in other eukaryotes, PCD in yeasts can be induced by stressors, signalling molecules, and aging process [33–35], Furthermore, the test of PCD in yeasts can be facilitated by an already demonstrated assay using over-expressed Ate1 with clear and robust readouts [9]. Here, we found that exposure to oxidative stressors lead to Ate1 translocation to mitochondria and cell death induction with characteristics consistent to apoptosis. Furthermore, Ate1-mediated PCD requires the opening of the mitochondrial permeability transition pore (mPTP), leading to mitochondrial outer membrane permeabilization (MOMP), and the presence of mitochondrial apoptogenic protein Aif1. However, mitochondrial Ate1 does not appear to directly induce ETC alterations or increase in ROS generation. Vice versa, ETC activity and ROS are not directly required for the execution of Ate1-indcued PCD. Finally, the execution of Ate1-mediated cell death is largely independent of most cytosolic PCD-relevant mechanisms, including UPS, autophagy, and ER-UPR. Therefore, our data suggests that Ate1 is an ancient and conserved regulator of the mitochondrial cell death pathway that was previously overlooked.

## Results

### Ate1 is enriched in mitochondria upon stress treatments and induces cell death

**Fig. 1:**
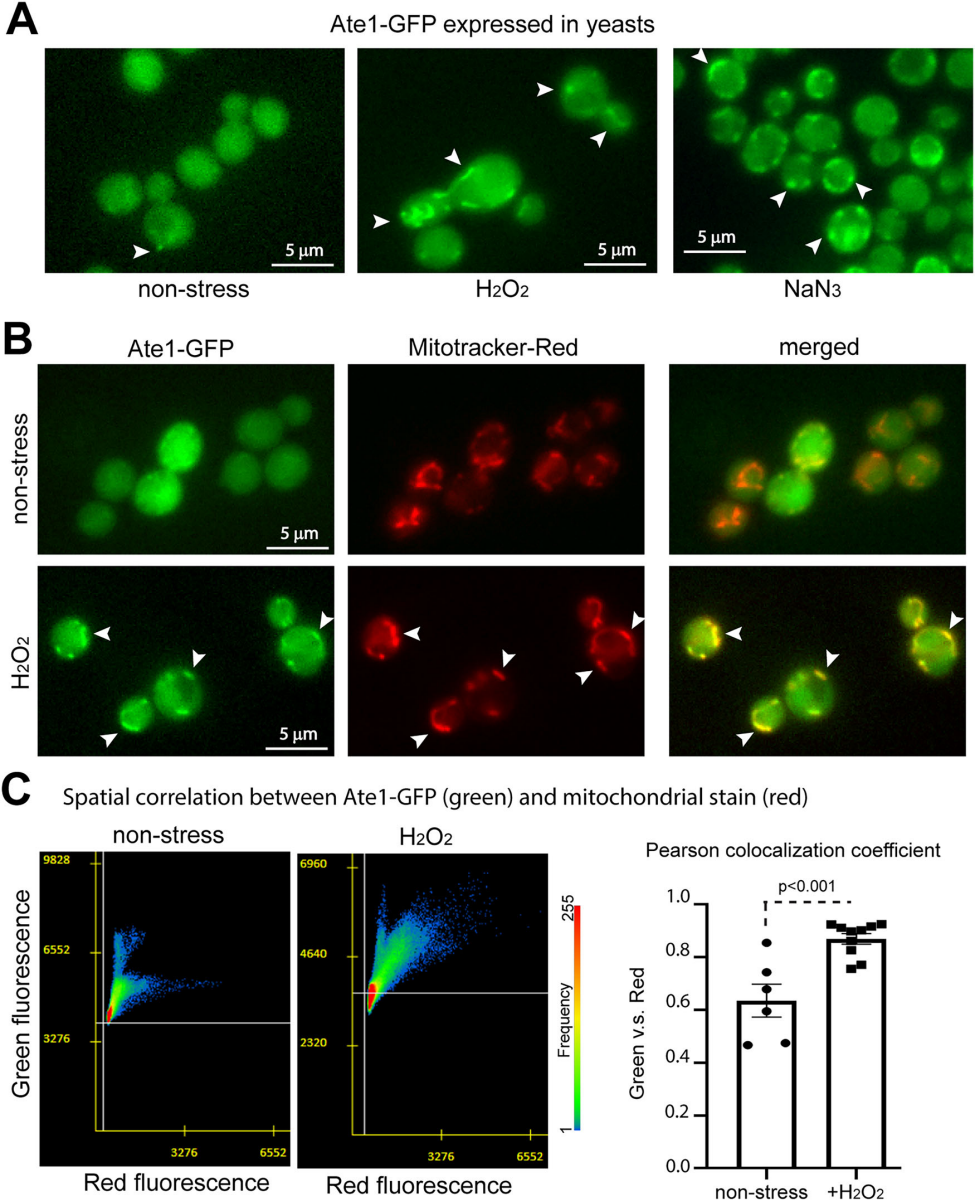
Visualization of Ate1 colocalization with mitochondria upon treatments of oxidative stressors. **A** Representative fluorescence microscopic images showing the signal of Ate1 tagged with a C-terminal GFP, which is driven by the endogenous *ATE1* promoter at the native chromosome locus. The yeast cells were either treated with 5 mM H_2_O_2_ or 5% NaN_3_, or not treated with anything (nonstress). The observation started promptly within 5 min of treatments. The BY4741 strain yeast was used for tests in all figures unless otherwise indicated. **B** The above-mentioned Ate1-GFP expressing yeast cells were treated with stressors H_2_O_2_ (5 mM) and stained with mitochondria-specific dye Mitoracker-Red. Arrow point to several locations where obvious colocalization of the green and red fluorescent signals are observed. **C** To quantify the mitochondrial translocation of the above-mentioned Ate1 under stressing conditions, Pearson colocalization coefficient analysis was used to measure the relative colocalization of GFP tagged Ate1 and mitochondria-specific dye (Mitotracker-red) in non-stressing and stressing conditions, using randomly selected microscopy images for regions of interest contains at least 50 cells for each (*n* = 6 and 10 for the non-treated and H_2_O_2_ treated groups). As in elsewhere in this study, unless otherwise indicated, the error bars represent standard deviation (S.D.), and the *p* values were calculated by two-tailed student *t*-test.

In our previous study, we observed a trace amount of Ate1 protein located inside the mitochondria in both yeast and mammalian cells. As a first test to see how this localization is related to cell death, we examined the distribution of Ate1 in the presence or absence of oxidizing chemicals, including hydrogen peroxide (H_2_O_2_) and sodium azide (NaN_3_), which are potent agents to induce PCD. To facilitate the tracing, the endogenous Ate1 in the yeast genome was fused with a GFP tag, which by itself does not appear to affect the function or location of Ate1 as shown in previous publications from us and others [9, 17, 36]. In absence of a stressor, the signal of Ate1 appears to be largely diffuse, as detected by microscopy. However, upon the exposure to oxidizing agents (H_2_O_2_ or NaN_3_), the Ate1-GFP signal rapidly enriches to puncta-like structures within 10 min (Fig. 1A). These structures colocalize with the Mitrotracker-Red signal, a marker for mitochondria (Fig. 1B), suggesting that Ate1 translocate to mitochondria in response to oxidative stress. To further quantitatively validate this change in Ate1 localization upon stress, we measured the Pearson colocalization coefficient of the Ate1-GFP and mitochondrial signals by microscopy. We found significant increased colocalization in stressor-treated cells compared to the non-stressed cells (Fig. 1C).

**Fig. 2:**
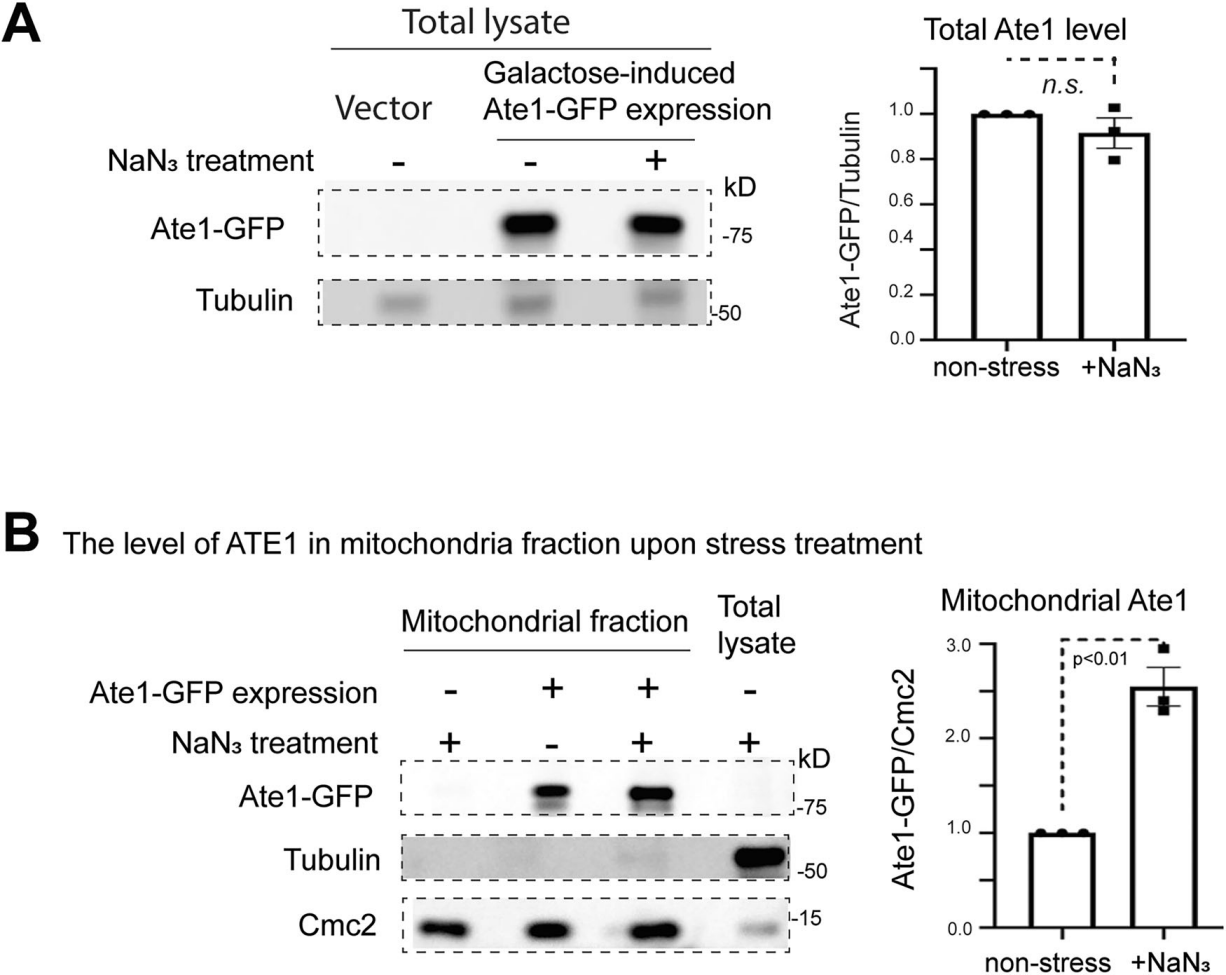
Oxidative stress induced by NaN_3_ increases mitochondrial Ate1 localization. **A** Left panel: representative Western blot (WB) showing total levels of Ate1-GFP in W303 wild-type strain treated either with or without 5%NaN_3_ for 30 min. The Ate1-GFP was cloned in a pYES2 vector with Galactose-inducible promoter (pGAL). The same construct was used elsewhere in this study unless otherwise indicated (such as in Fig. 1). To avoid acute cell death, the expression was induced by switching to culture media containing a moderate concentration of galactose (0.5%) and incubated for 3 h at 30 °C. Empty vector serves as a negative control. Alpha tubulin is used for protein loading control. Right panel: quantification of the total Ate1 levels in the cells treated with or without 5%NaN_3_ for 30 min with alpha tubulin as the loading control and normalized to the group without the NaN_3_ treatment (*n* = 3). **B** Left panel; representative Western blot showing Ate1-GFP levels in purified mitochondrial fractions, which was treated with proteinase K (5 μg/ml) to remove proteins that are not protected by the mitochondrial membrane. Alpha-tubulin is used as a marker for cytosolic protein contamination, while mitochondrial intermembrane space protein Cmc2 serves as a marker for mitochondrial proteins. Right panel: showing the quantification of mitochondrial Ate1-GFP levels normalized to mitochondrial protein Cmc2 (*n* = 3).

To corroborate the microscopy-based observations, we utilized an ectopic expression of a GFP-tagged recombinant Ate1 driven by a galactose-inducible promoter. The over-expressed Ate1 has been shown to increase cellular sensitivity to stressors and directly induce PCD [9, 37]. To focus on the early event of PCD, the cells were analyzed at 3 h post-induction, at which more than half of the cells are anticipated to be viable [9]. We then isolated the mitochondrial fraction from these cells treated (or not) with the oxidative stressor NaN_3_. By using proteinase K to remove proteins attached to the mitochondrial outer membrane and by immunoblotting, we examined the level of Ate1-GFP in the whole cell and the mitochondrial fraction. We observed that, with the comparable levels of overall recombinant Ate1 in the whole cells (because the expression is driven by galactose induction) (Fig. 2A), the mitochondria-located fraction of Ate1 is significantly increased in the stressed cells (Fig. 2B).

**Fig. 3:**
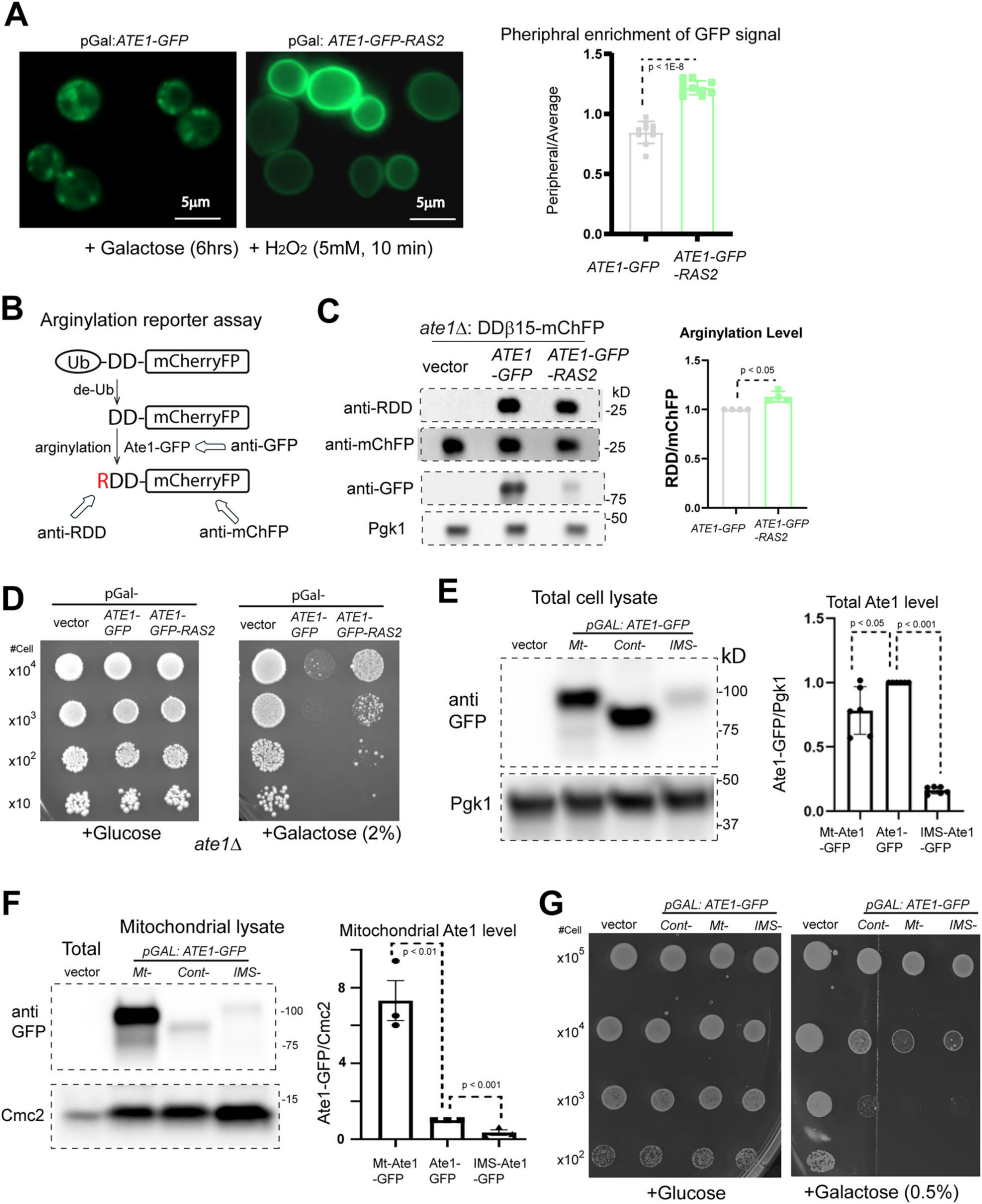
The mitochondrial translocation of Ate1 is essential for the cell death induced by Ate1 over-expression. **A** The left panel shows yeast cells containing Ate1-GFP and Ate1-GFP-Ras2, which were allowed to express for 6 h with 2% galactose induction and then briefly treated with oxidative stressor H_2_O_2_ for 10 min before the microscopic images were taken. The regular Ate1-GFP forms puncta-like structures, similar as shown in Fig. 1, while Ate1-GFP-Ras2 remains localized to the periphery of the cells. The right panel shows quantitative measurement of the distribution of the GFP signal. This was done by calculating the ratio of the mean GFP signal from the plasma membrane (using profile method on Zeiss ZenBlue software; stroke = 1 pixel width) versus the mean GFP throughout the whole cell (on 10 randomly chosen individual yeast cells). Homoscedasticity was determined via Levene’s test, before being analysed via a two-tailed Student’s *t*-test (*n* = 10). Error bars represent S.D. **B** Scheme illustrating the principle of the reporter for the arginylation activity inside yeast cells. The N-terminal ubiquitin domain of the reporter protein DD-β15-mCherryFP will be promptly removed by endogenous de-ubiquitination (de-Ub) enzymes in the cell, exposing the penultimate peptide DD-β15, which is derived from the N-terminus of mouse β-actin and is known to be arginylated in vivo [9, 39]. The arginylated N-terminus can be recognized with a specific antibody anti-RDD [9]. Antibodies for mCherryFP (mChFP) and GFP can be used to probe the levels of the reporter protein and the GFP-fused ATE1, respectively. **C** To test the arginylation activity of different forms of Ate1 (Ate1-GFP or Ate1-GFP-Ras2), they were expressed in *ate1*Δ yeast (to avoid the interference of endogenous ATE1), which was also simultaneously expressing the reporter protein DD-β15-mCherryFP. The arginylation level of the reporter protein was measured as described in (**B**). To avoid potential carryover of antibody signals, the same set of samples were loading twice in different gels for the probing of anti-RDD and anti-mCherry, separately. Pgk1 serves as loading controls for the yeast proteins. The left panel shows representative WB images while the right panel shows quantification from multiple repeats (*n* = 4). **D** Growth test of *ate1*Δ yeast cells carrying either the empty expression vector, or the one containing Ate1-GFP or Ate1-GFP-Ras2 was conducted by a serial dilution growth assay on either plate containing glucose or galactose, where the expression of Ate1 is not induced or induced, respectively. **E** The left panel shows representative Western blots showing the expression of different Ate1 constructs in total cell lysate. The level of Ate1 was probed by its GFP fusion tag and Pgk1 was used as a loading control. The employed Ate1 constructs include the original Ate1-GFP as control (labelled as “cont”), and the forms that are expected to be targeted to the mitochondrial matrix (Mt) or mitochondrial intermembrane space (IMS). The IMS- and Mt- targeting sequences were derived from *S. cerevisiae* Cytochrome b2 (Cyb2) with or without a deletion of a 19 amino acid(Aa) stop-transfer transmembrane signal as described in published studies [40, 41, 74]. To avoid disrupting mitochondria, the expression of these different Ate1 constructs was achieved by adding 0.5% galactose and incubated for 3 h at 30 °C. The signal of the GFP-fused Ate1 were probed by anti-GFP with Pgk1 as loading controls for total proteins. The right panel shows quantification (*n* = 6). **F** Similar to (**E**), except that the mitochondrial-specific fractions, treated with proteinase K to remove any proteins attached on the outside, were used to prepare the lysates for the measurement of the levels of Ate1 inside mitochondria, with Cmc2 as the loading controls for mitochondrial proteins. The right panel shows quantification (*n* = 3). **G** Growth test of WT W303 yeast cells carrying either the empty expression vector, Ate1-GFP (cont-), the matrix localized Ate1-GFP (Mt-), or the IMS located Ate1-GFP (IMS-) by a serial dilution growth assay on either plate containing galactose or glucose for the induced expression (or not) of Ate1. Note that the concentration of galactose (0.5%) is lower and the cell loading were higher than elsewhere to allow the display of the difference between the different forms of Ate1. Plates were incubated at 30 °C and images were taken after 3 days.

To further test if the mitochondrial localization of Ate1 is required for the induction of cell death, we over-expressed Ate1 for an extended duration, a condition that can cause PCD in the absence of exogenous stressor [9]. As a control, to prevent Ate1 mitochondria localization, we fused the Ate1-GFP with an additional tag derived from the Ras2 protein, which is expected to be conjugated to lipid molecule on the plasma membrane [38]. As validated by microscopy, the expressed Ate1-GFP-Ras2 localizes to the periphery of the cells even under stress, and does not form any puncta-like structures (characteristic of mitochondria) unlike Ate1-GFP (Fig. 3A). To test if the Ras2 tag negatively affects the activity of Ate1, we used an arginylation reporter, DD-β15, which is derived from the N-terminal sequence of a known arginylation substrate β-actin [9, 39]. It is fused to a fluorescence protein mCherryFP, which is expected to be diffusively distributed in the cytosol and nucleus (Fig. 3B). By using the co-expression of the reporter with the recombinant Ate1, we found that, while a lower expression level of Ate1 is seen with the Ras2-tagging, the arginylation level of the reporter remains similar (Fig. 3C). This indicates that the Ras2 tagging did not decrease the arginylation activity of the recombinant Ate1 inside the cytosol. However, in contrast to the original Ate1-GFP, the overexpression of Ate1-GFP-Ras2 leads to a higher viability of yeast cells (Fig. 3D), suggesting that blocking the mitochondrial localization of Ate1 minimizes its effects in inducing cell death. To test this possibility from a reverse angle, we tagged the Ate1 with mitochondrial targeting signals [40, 41] to either the intermembrane space (IMS) or matrix (Mt) (Fig. 3E, F). We found that, while the IMS- and Mt-targeted Ate1 have lower amount of overall expression levels compared to the original Ate1, they exhibited more potent effects for inducing cell death (Fig. 3G). Therefore, our results together showed that the mitochondrial localization of Ate1 is required for Ate1-induced cell death.

### Ate1 induces cell death with characteristics of apoptosis and a dependency on mitochondrial permeability transition pore

**Fig. 4:**
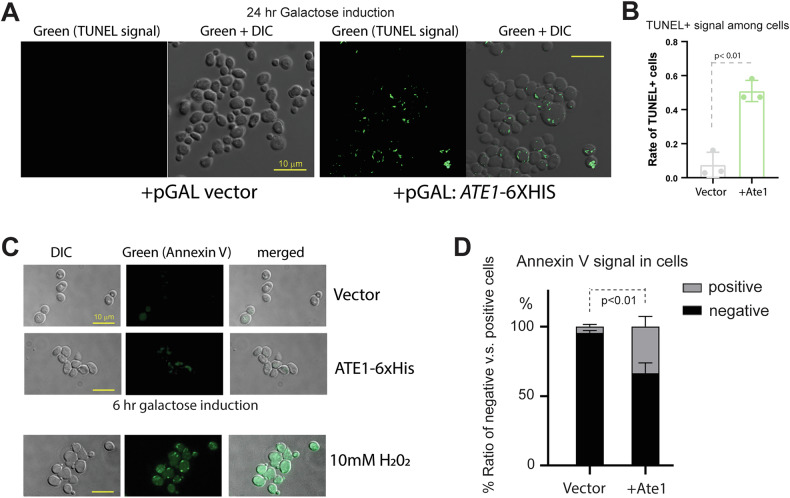
Ate1-overexpression triggers cell death events with characteristics of apoptosis. **A** Representative microscopy images displaying the presence of DNA fragmentations probed by the TUNEL assay. The *ate1*Δ yeasts were transformed with a plasmid vector (pYES2) containing Ate1 driven by a galactose-inducible promoter (pGAL). To avoid introducing additional fluorescence, a C-terminal 6 × His tag was used and the construct is termed (pGAL: ATE1-6 × His). As a control, the empty vector was used. The induction was performed by switching to a selection media containing 2% galactose for 20 h (hr, or h). The panels on the left display only the TUNEL signal (green fluorescence), while the panels on the right are an overlay of TUNEL and Differential Interference Contrast (DIC) microscopic images. The yellow scale bars represent a length of 10 μm, which is similarly used in other images in this study unless otherwise indicated. **B** Quantification of the frequencies of TUNEL-positive cells described in (**A**) based on 3 randomly selected (*n* = 3) microscopy images with at least 200 cells in each image. **C** Similar as (**A**), except that fluorescently labelled Annexin-V (green) was used to stain cells with apoptotic signs, and that the wild-type yeasts were used. On the top panel are yeasts with the empty vector or Ate1-6 × His, which were induced by 2% galactose for 6 h. On the bottom, yeasts treated with 10 mM H_2_O_2_ for 200 min was used as a positive control for apoptotic signals. **D** Quantification of the percentage of cells described in (**C**) that are negative or positive of Annexin V signals. The analysis is based on 3 randomly selected microscopy images (*n* = 3) with at least 70 cells in each image.

Similar to many other eukaryotes, yeasts possess multiple PCD pathways such as apoptosis, necrosis, and macro-autophagy, all of which are directly or indirectly influenced by mitochondria [42]. To determine the nature of the Ate1-induced PCD, we first used the TUNEL assay to examine yeasts with 20-h galactose-induced overexpression of Ate1 (fused with a non-fluorescence tag 6 × His). We found the induced Ate1 overexpression is accompanied with positive signs of large-scale DNA cleavages as shown in the TUNEL assay (Fig. 4A, B), which is a hallmark of apoptosis (for the execution phase, a mid-stage of apoptosis) [43]. Consistently, the examination of the appearance of Annexin V staining, a marker for the initiation phase of apoptosis, showed that the over-expression of Ate1 leads to significantly increased numbers of Annexin V positive cells at the early timepoint (6 h) (Fig. 4C, D).

**Fig. 5:**
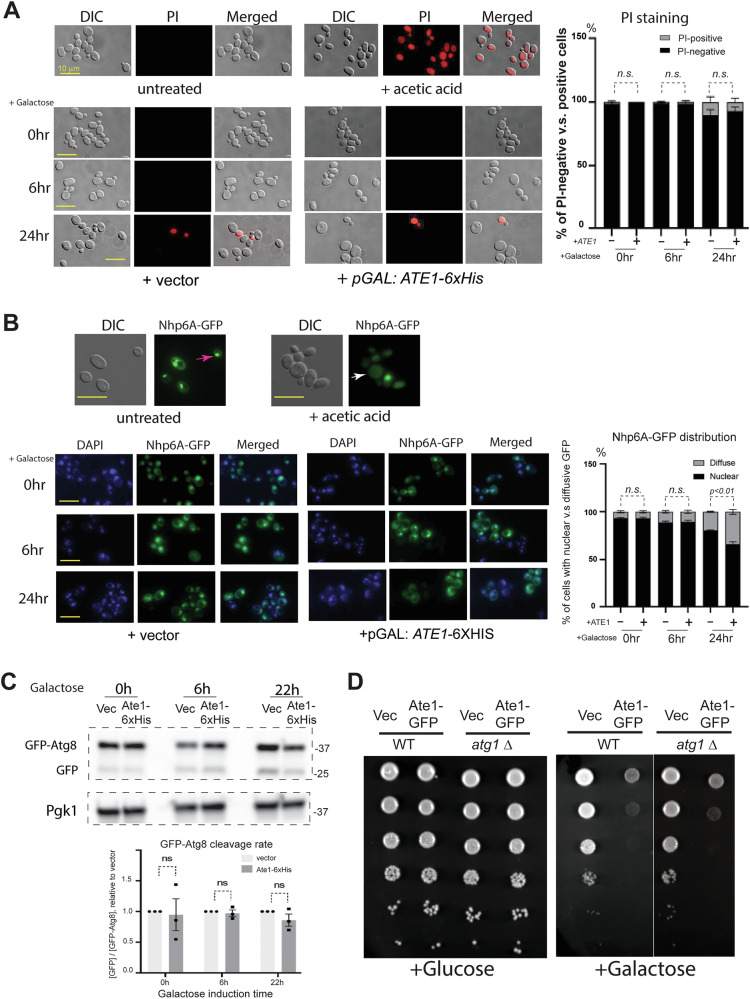
Ate1-overexpression have no major effects on necrosis or autophagy. **A** Top panel displays representative cropped microscopy images of DIC channel showing the location and morphology of the yeasts, the red fluorescence channel showing the signal of Propidium iodide (PI) staining, or the merged images. The BY4741 WT yeasts were either untreated or treated with 200 mM acetic acid for 200 min as a positive control to induce necrosis. Bottom panel displays corresponding images of ate1∆ cells harbouring Ate1-6 × His in the pYES2 vector, which is driven by a galactose-inducible promoter (“pGAL: *ATE1*-6 × His”). As a control, the empty vector (“pGAL”) was used. The induction was performed with 2% galactose for 6 h, 24 h, or not induced (0 h). The rates of PI-positive and negative cells in these cells are shown in the bar graph on the right side, which was calculated based on analysis of 3 randomly chosen microscopy images (full-size, uncropped) for each sample groups (*n* = 3). Each image contains at least 100 cells. Error bar denotes standard error of means (SEM). **B** Similar as in (**A**) except the BY4741 wild type yeast cells harbouring a constitutive expression vector for Nhp6A-GFP (PESC-LEU-NHP6A-GFP) was used. On the top panel, the pink arrow in the untreated cells indicates a representative location where the Nhp6A-GFP is enriched in a focal point. The white arrow in the acetic acid treated cells points to a cell where the Nhp6A-GFP appears to be diffusive, indicating necrosis. On the bottom panel, the Nhp6A-GFP containing cells are co-transformed with either the galactose-inducible Ate1 (pGAL: Ate1-6 × His) or the empty vector (pGAL). The induction with 2% galactose was performed for either 6 or 24 h or not (0 h). Yeast nuclei were stained with DAPI. The graph on the right side shows percentages of cells showing nuclear localization or diffusive distribution of NHP6A-GFP, which was calculated from 3 randomly chosen microscopy images (full-size, uncropped) for each sample groups (*n* = 3). Each image contains at least 100 cells. Error bar denotes standard error of means (SEM). **C** Top panel: representative WB images of wild-type yeast cells (BY4741) carrying the autophagic reporter GFP-Atg8 either with empty plasmid vector (vec) or the one carrying galactose-inducible Ate1-6 × His. The induction was initiated by switching from raffinose-containing media (0 h) to 2% galactose-containing media for 6 or 22 h. The levels of the full-length GFP-Atg8 and the cleavage product (GFP) were probed by anti-GFP while Pgk1 was used as a loading control. The graph on the bottom panel shows the fold differences in cleavage ratio between the full-length GFP-Atg8 and the cleaved GFP (*n* = 3). See also Suppl Fig. S1A for the positive control of yeast treated with rapamycin to induce a high level of cleavage on GFP-Atg8. **D** Growth test of WT or *atg1*Δ yeast cells carrying either Ate1-GFP driven by the galactose promoter or the empty vector (“Vec”) by a serial dilution growth assay on either plate containing galactose or glucose. Note that the growths of *atg1*Δ and the WT yeasts have intrinsic difference on the galactose media. Therefore, to accurately compare the growth difference specifically induced by Ate1, the exposures of these two strains were taken differently so that the cells with the vector control show similar growth.

Next, we examined the role of Ate1 in necrosis induction by using Propidium iodide (PI) to label cells with compromised peripheral membranes, which takes place during early phase of necrosis or the late (degradation) phase of apoptosis. We found that Ate1 overexpression did not lead to significant increases of PI-positive cells at either 6-h or 24-h timepoint, compared to the vector control (Fig. 5A). To further corroborate with this result, we employed GFP conjugated Nhp6A [44]. This protein is normally enriched in the nucleus and a redistribution from nucleus to the cytosol indicates nuclear envelope disturbance, which takes place during late phase necrosis in yeast [45]. We found that Ate1 over-expression, compared to the vector control, did not significantly increase the frequency of Nhp6A diffusion at the 6-h timepoint (Fig. 5B). At the later timepoint (24 h), elevated Nhp6A was seen in both vector control and Ate1 over-expressed groups, although the Ate1 over-expressed group have a much higher value (Fig. 5B). This is likely due to multiple factors including the stress associated from switching the culture media, considering that a time-dependent increase of Nhp6A diffusion was also observed in the vector control. While Nhp6A diffusion is usually considered a sign for necrosis, it can also be caused by apoptosis, which at the execution phase leads to nuclear envelope disruption [45]. The later possibility is further consistent with the TUNEL signal observed at the 24 h timepoint. However, the distinction of these two possibilities is challenging because mid-late stages of apoptosis often share characteristics with necrosis. Nevertheless, increases in positive PI staining or Nhp6A diffusion were not observed in Ate1 overexpressed cells at the earlier timepoints (such as 6 h) when signs of early apoptosis (Annexin V staining) were present.

Finally, to test the signs of macro-autophagy, we first examined the cleavage of GFP-fused Atg8, an established marker for autophagy (Suppl Fig. S1A). We found that the over-expression of Ate1 did not increase the cleavage of GFP-Atg8 in the examined timepoints (up to 22 h) (Fig. 5C). Consistently, the deletion of *ATG1*, a gene essential for the initiation of autophagy, did not generate any obvious suppression effects on the cell death caused by Ate1 induction (Fig. 5D).

Therefore, the evidence we gathered suggests that, under the conditions tested in this study, overexpressed Ate1 preferentially triggers apoptosis at least in the initial stage. Considering the known biological significance of apoptosis in physiological and pathological conditions in eukaryotes [46], we chose to focus on this particular PCD process.

**Fig. 6:**
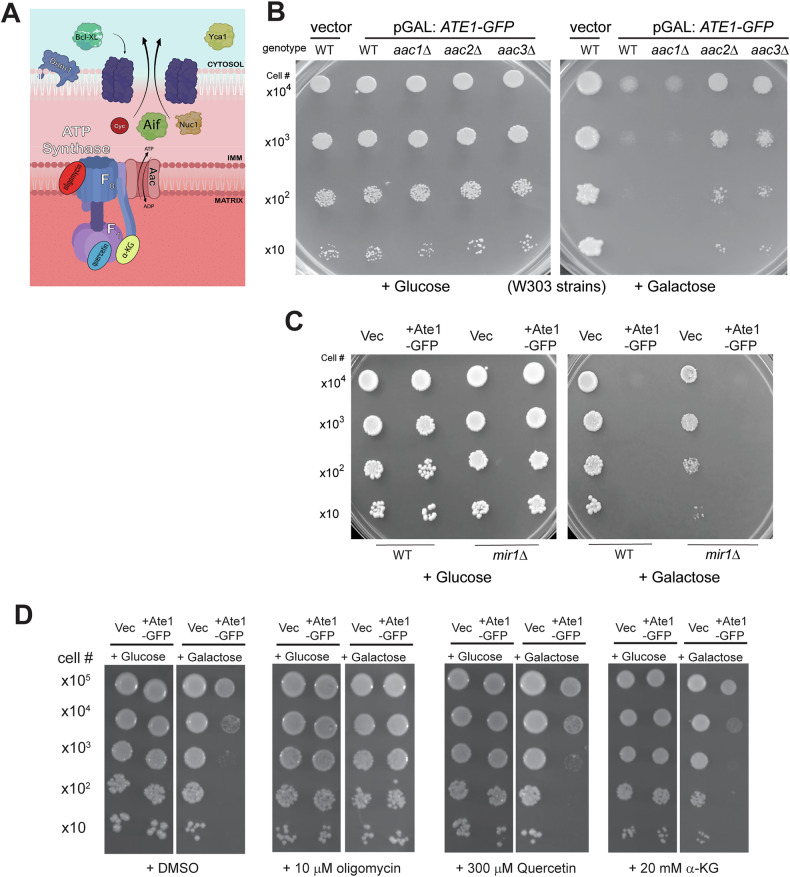
Ate1-induced cell death involves mitochondrial permeabilization transition pore. **A** A diagram showing some of the key components in yeasts that affect mitochondrial permeabilization transition pore (mPTP) and apoptosis. Note that the Bcl-XL is not an endogenous protein of yeast, but it can interact with the mitochondrial outer membrane permeabilization (MOMP) event [56]. **B** Growth of yeast cells (WT, *aac1*Δ, *aac2*Δ¸ *aac3*Δ, all on W303 strains) carrying either the empty expression vector (vector) or *pGAL1:ATE1-GFP* (+Ate1-GFP) was measured by a serial dilution growth assay on either plate containing 2% glucose or 2% galactose, where the expression of Ate1 is not induced or induced, respectively. Plates were incubated at 30 °C and images were taken after 2–3 days. **C** Similar to (**B**), except that WT and *mir1*Δ yeasts were used. **D** Serial dilution growth assay to assess changes in growth of WT yeast induced with pGAL: Ate1-GFP in the context of various ATP synthase inhibitors. Yeasts were grown in raffinose-containing liquid media before being washed, serially diluted in H_2_0, and plated to either glucose or galactose-containing plates with the designated concentrations of inhibitors, including Oligomycin A, Quercetin, and α-ketoglutarate (α-KG). Plates were allowed to grow three days before images were taken. DMSO (at a final concentration of 0.004% in the plate) was used as vehicle control. See also Suppl Fig. S1B to see the equal growth rate of the involved mutant yeast strains carrying the empty vector on galactose-containing media, compared to the WT strain.

In most eukaryotes, the mitochondrial apoptotic pathway is driven by the formation of the mPTP, a large, unselective channel present within the inner membrane, which leads to the swelling of mitochondria and rupture of the mitochondrial outer membrane. It is important to point out that the molecular composition of the mPTP has shown a degree of flexibility. However, some of the most conserved core elements include the F_o_ module of the F_o_F_1_-ATPase and isoforms of the adenine nucleotide transporter (ANT) (Fig. 6A) [29, 47–49]. To determine the involvement of these components in Ate1-induced apoptosis, we overexpressed Ate1 in yeast strains with deletions of the corresponding genes. We found that the deletions of two ANT isoforms (*AAC2* and *AAC3*) were able to generate significant rescue effects on the Ate1-induced lethality (Fig. 6B and Suppl Fig. S1B). In addition to mPTP formation during apoptotic events, ANT normally facilitates the exchange of nucleotides (ATP/ADP) coupled with ATP synthesis. To exclude the possibility the later role is involved in Ate1-induced cell death, we examined the knockouts of *MIR1*, which is involved in mitochondrial phosphate transport (important for ATP synthesis) but not directly involved in mPTP formation [29]. We found that this gene knockout does not generate rescue effects on cell death induced by Ate1 overexpression (Fig. 6C and Suppl Fig. S1B). To corroborate with the above genetics-based tests, we used specific inhibitors to block the functions of F_o_F_1_-ATPase, another important component of mPTP. We found that the application of oligomycin, which specifically blocks the function of the F_o_ subunit of F_o_F_1_-ATPase [50], potently supresses the lethal effects of Ate1-overexpression (Fig. 6D). However, in addition to blocking the formation of the mPTP, oligomycin also would compromise ATP synthesis. To exclude the latter possibility, we applied two different reagents, quercetin and α-ketoglutarate, which inhibit the F1 compartment of ATPase to impair ATP synthesis but not the mPTP formation [51, 52]. We found these treatments have little effects on the Ate1-induced cell death (Fig. 6D). Together, these data suggest that Ate1 specifically regulates the mitochondrial mPTP formation for the induction of apoptosis.

**Fig. 7:**
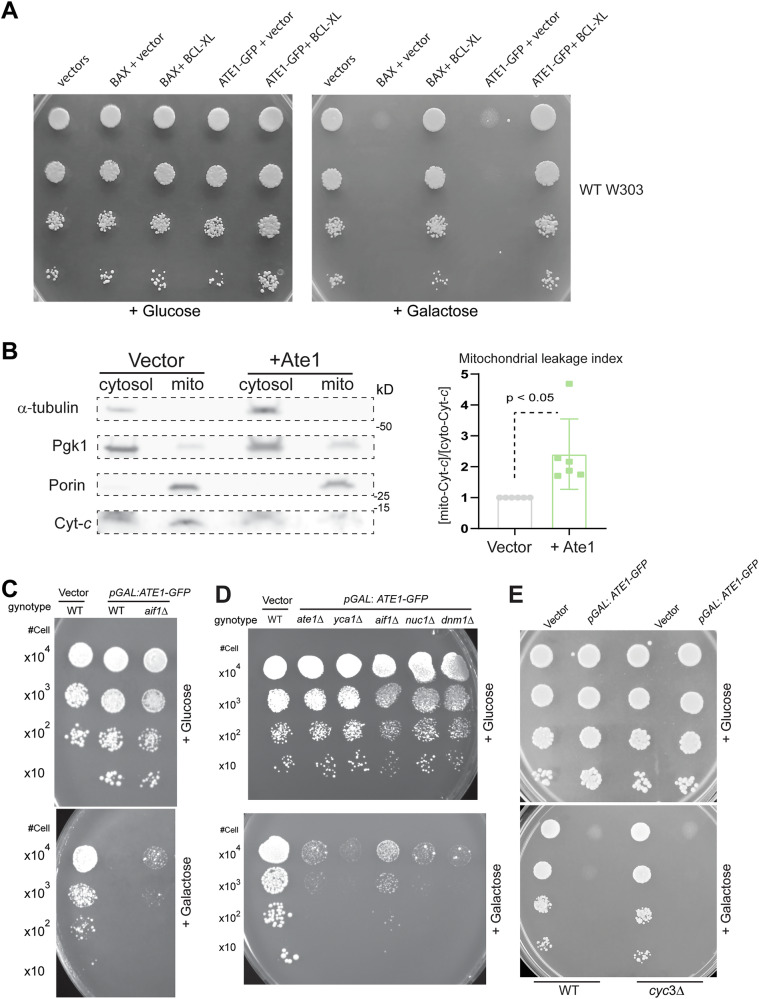
Ate1-induced cell death involves mitochondrial outer membrane permeabilization. **A** Growth of yeast cells (W303 strain, WT) carrying either the empty expression vectors (pYES2-URA3 and pBF339-TRP1 vectors), or the galactose-inducible mouse BAX (pBM272-pGAL-*BAX*-URA3) and yeast Ate1 (pYES2-pGAL-*ATE1-GFP*-Ura3) in the presence of constitutively expressing Bcl-xL (pBF339-ADH-*BCLxL*-TRP1) or the vector (pBF339-TRP1) was measured by a serial dilution growth assay on either plate containing 2% glucose or 2% galactose, where the expression of Ate1 or BAX is not induced or induced, respectively. Plates were incubated at 30 °C and images were taken after 3 days. **B** Representative Western blots displaying changes in the distribution of cytochrome c (Cyc) between mitochondria and cytosol in yeast cell where Ate1-6 × His was induced for expression (“+Ate1”) with 2% galactose in Ura-minus liquid media for 6 h at 30 °C. The separated cytosolic fraction and mitochondrial fraction were compensated by buffer to be equal volume before analysis. Alpha-tubulin and Porin (VDAC) were used as markers to display the purity of cytosolic and mitochondrial fractions, respectively. Pgk1 was used as an additional loading control. The quantification of the blot was based on *n*= 6; error bars represent S.E.M. **C** Growth of yeast cells (WT or *aif1*Δ) carrying either the empty expression pYES2 vector (“Vector”) or pYES-pGAL1:*ATE1-GFP* (“+Ate1-GFP”) was measured by a serial dilution growth assay on either plate containing 2% glucose or 2% galactose, where the expression of Ate1 is not induced or induced, respectively. Plates were incubated at 30 °C and images were taken after 3–5 days. **D** Similar to (**C**), except that WT, *aif1*Δ, *ate1*Δ¸ *yca1*Δ, *nuc1*Δ, and *dnm11*Δ) yeasts were used. **E** Similar to (**C**), except that WT and *cyc3*Δ yeasts were used. See also Suppl Fig. S1C to see the equal growth rate of the involved mutant yeast strains carrying the empty vector on galactose-containing media compared to the WT strain, if they were not already shown in this figure.

The formation of the mPTP is usually further connected with the MOMP events, which leads to the release of apoptogenic proteins such as cytochrome *c* (Cyc), apoptosis-inducing factor (Aif1), and Endonuclease G (Nuc1) from the mitochondrial intermembrane space to the cytosol/nucleus, where they contribute to the apoptosis execution. To test the relevance of MOMP in Ate1-induced cell death, we utilized exogenous expression of mammalian Bcl-xL, a Bcl-2 family protein that is known to block the outer channel of MOMP formed by both Bax-dependent [53] and independent mechanisms [54, 55]. Although yeast lacks Bcl-2 family proteins, it contains the other mPTP and MOMP components that were shown to react to mammalian Bcl-2 in a similar fashion [56]. To test the possibility that Ate1-induced MOMP can be prevented by Bcl-xL, we co-expressed this protein in yeast cells with the over-expressed Ate1. We found that Bcl-xL expression indeed has measurable effects in antagonising Ate1-induced lethality, similar to its effect found in mammalian Bax-induced yeast cell death (Fig. 7A). To directly test if over-expressed Ate1 causes MOMP, we examined the distribution of Cytochrome *c* (Cyt-*c*) between mitochondria and the cytosol. Indeed, we found a higher portion of Cyt-*c* present in the cytosol versus (*v.s*.) mitochondria in yeast cells overexpressing Ate1 (Fig. 7B), indicating a leakage of mitochondrial contents. Furthermore, as another validation of the relevance of MOMP events in Ate1-induced cell death, and to delineate the role of different mitochondria-containing apoptogenic factors, we used yeast with deletions of those corresponding genes, including *AIF1* and *NUC1*. Considering yeast contains two *CYC* genes, we used the deletion of *YCA1*, which codes for the metacaspase that is supposed to respond to Cyt-*c* release. We also used the deletion of *CYC3*, which codes for the enzyme responsible for Cyt-*c* maturation by mediating heme linkage. We found that, while we observed no significant rescue by the deletions of *YCA1* (Fig. 7D), *CYC3* (Fig. 7E), or *NUC1* (Fig. 7D), *AIF1*-knockout generated measurable rescue effects on Ate1-induced cell death (Fig. 7C, D), which supports the role of Ate1 in MOMP as a part of the mechanism of Ate1-mediated PCD. In comparison, the deletion of Dynamin-related GTPase (*DNM1*), which typically mediates mitochondria fission and receives signal from the cytosol and the cell membrane to trigger apoptosis, shows no effects (Fig. 7D). See also Suppl Fig. S1C for the equal growth capacity of the employed yeast stains in galactose-containing media. Therefore, our data together suggest that the elevation of Ate1 induces apoptosis by an intrinsic, mitochondria-dependent pathway.

### Mitochondrial electron transport chain (ETC) and mitochondria-generated reactive oxygen species (ROS) are not directly required for the execution of Ate1-induced PCD

Mitochondrial ETC activity and mitochondria**-**generated ROS have been shown to play important role in yeast apoptosis. Recent studies showed that an Ate1-deletion in either yeast or mammalian cells lead to a decrease in aerobic respiration [31, 57]. This prompted us to test if an elevation of Ate1 induces a hyperactivation of mitochondrial respiration and/or ROS generation, which then can lead to apoptosis by other mechanisms including the activation of caspases.

**Fig. 8:**
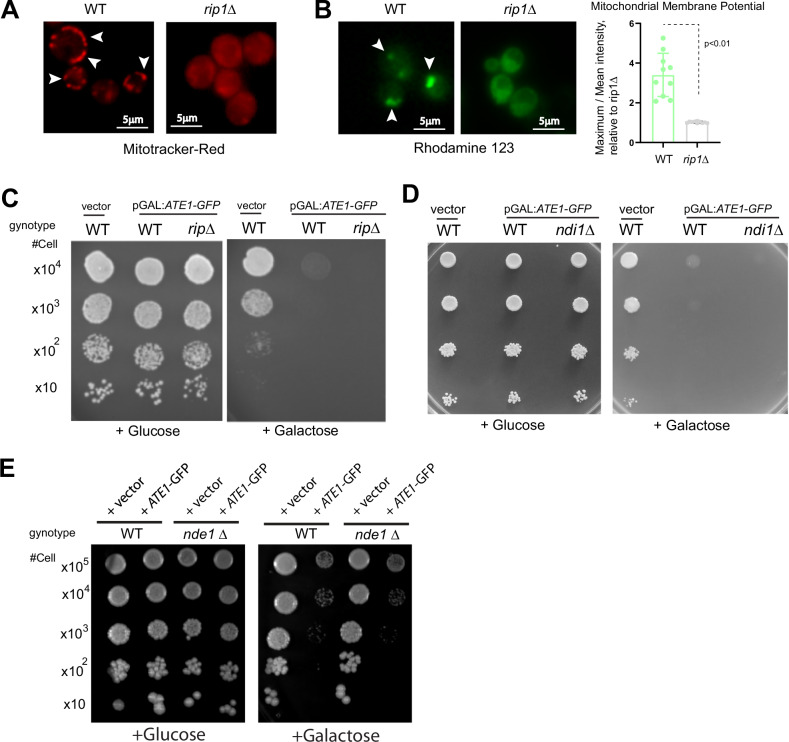
The mitochondrial respiratory activity is not directly required during Ate1-driven cell death. **A** The mitochondrial membrane potentials in yeast cells, WT or *rip1*Δ, were probed by staining dyes Mitotracker-red (red). White arrows point to representative locations with high intensities. **B** Left panel shows representative microscopy images of mitochondrial membrane potentials in WT or *rip1*Δ yeasts measured by rhodamine 123 (green). White arrows point to selected locations with high intensities. Right panel shows the corresponding quantification. The relative membrane potential was calculated by dividing the maximum fluorescence intensity by the mean intensity. The data was determined as heteroscledastic via Levene’s test, before being analysed via student’s *t*-test. 10 randomly chosen yeast cells were included in each group (*n* = 10). **C** Growth of yeast cells (WT or *rip1*Δ) carrying either the empty expression vector pYES2 (“Vector”) or pYES2-pGAL1:*ATE1-GFP* (“+Ate1-GFP”) was measured by a serial dilution growth assay on either plate containing 2% glucose or 2% galactose, where the expression of Ate1 is not induced or induced, respectively. Plates were incubated at 30 °C and images were taken after 3–5 days. **D** Similar to (**C**), except that *ndi1*Δ was used. **E** Similar to (**C**), except that *nde1*Δ was used. See also Suppl Fig. S1D to see the equal growth rate of the involved mutant yeast strains carrying the empty vector on galactose-containing media compared to the WT strain, if they were not already shown in this figure.

To test if the ETC activity is required for Ate1-induced cell death, we used a respiratory-deficient yeast strain due to the deletion of the RIP1 gene, which encodes for a catalytic subunit of complex III. While the deletion of RIP1 indeed affected mitochondrial membrane potential, as confirmed by two different membrane potential-dependent dyes (mitotracker-red and rhodamine 123), it did not rescue Ate1-induced cell death (Fig. 8A–C). To validate this result, we examined the knockout of *NDI1* and *NDE1,* which encodes for the mitochondrial internal NADH dehydrogenase, and mitochondrial external NADH dehydrogenase, respectively. As such, the deletion of these genes are expected to compromise ETC activity. Similar to the finding about *RIP1*-deletion, the knockout of *NDI1* or *NDE1* does not show any noticeable protection against Ate1-induced cell death (Fig. 8D, E). These results are consistent with the previously mentioned data regarding ATPase inhibition by α-ketoglutarate (α-KG) or quercetin (Fig. 6D), as well as the lack of effects by the deletion of *CYC3* (Fig. 7E), which is also essential for ETC function. Taken together, these data demonstrate that the execution of Ate1-mediated cell death does not directly requires ETC activity.

**Fig. 9:**
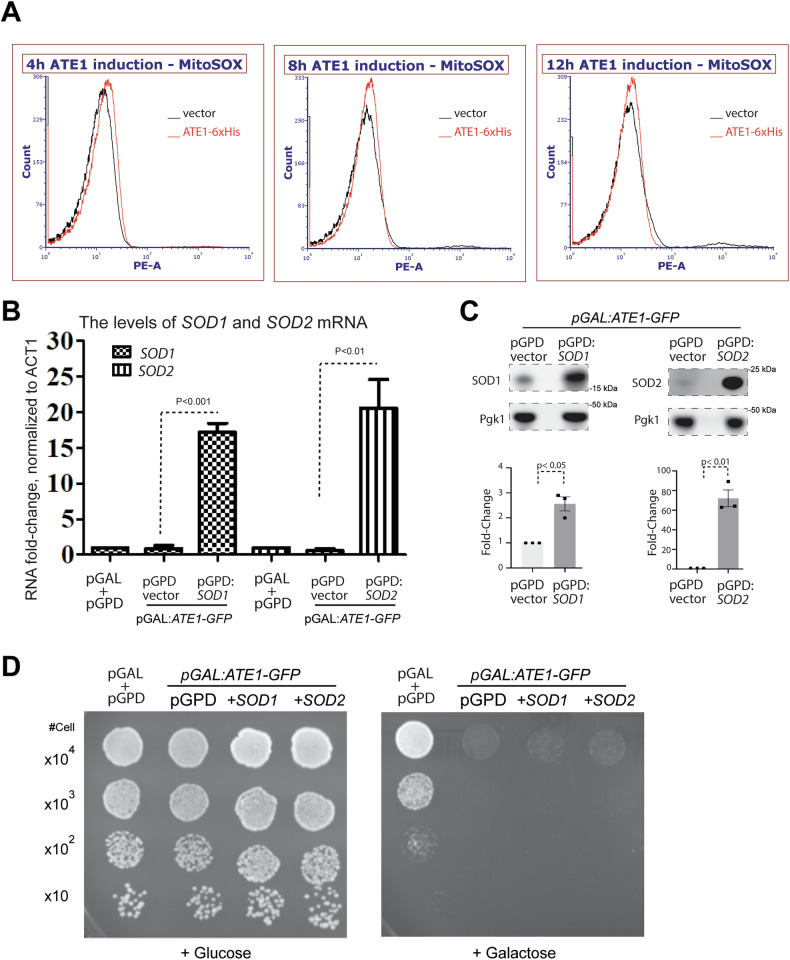
Mitochondrial ROS is not directly required during the Ate1-driven cell death. **A** Representative flow cytometry plots depicting mitochondrial ROS levels at indicated timepoints after galactose induction. The yeast cells contain either the empty vector control pYES2 (black) or the pYES2-*ATE1*-6 × His for the overexpression of the 6 × His tagged Ate1 (red). MitoSOX Red was used to stain for mitochondrial ROS, and samples were excited with a 488 nm blue laser and emission was measured using a 585 nm filter with a 42 nm bandpass. No obvious differences in mitochondrial ROS were noted between Ate1 and control samples, in any of the time points. **B** Graph shows the fold-changes of mRNA levels of yeast SOD1 and SOD2, measured by quantitative PCR in *ate1*Δ yeast cells carrying the combination of different vectors. These include the two empty expression vectors pYES2-URA (pGAL), and pGPD2-Leu2 (pGPD), the pYES2 vector containing Ate1-GFP (“pGAL:ATE1-GFP”), pGPD2 vector containing yeast SOD1 (“pGPD:SOD1”) or SOD2 (“pGPD:SOD2”). The loading was normalized by the level of *ACT1* mRNA. The fold-change was calculated relative to the group containing two empty vectors (*n* = 3). **C** Top panel shows representative WB images for the level of yeast SOD1 and SOD2, in *ate1*Δ yeast cells carrying a pYES2 vector containing Ate1-GFP (“pGAL:ATE1-GFP”) and the combination of either a pGPD2 vector containing yeast SOD1 (“pGPD:SOD1”) or SOD2 (“pGPD:SOD2”), or the empty vector pGPD2-Leu2 alone (“pGPD vector”). The yeasts were grown in the glucose-containing media, where the constitutive GPD promoter should be active. Pgk1 was used as a loading control. The fold-change was calculated relative to the group containing the pGPD vector (*n* = 3). **D** Growth of *ate1*Δ yeast cells carrying either the empty expression vectors pYES2-URA + pGPD2-Leu2 (vectors), pYES2-pGAL1:*ATE1-GFP* + pGPD2-Leu2 (n/a), or pYES2-pGAL1:*ATE1-GFP* in the presence of constitutively expression vectors of pGPD2-*SOD1*-Leu2 or pGPD2-*SOD2*-Leu2 (+*SOD1*, or +*SOD2*). The growth rate was measured by a serial dilution growth assay on Ura-minus, Leu-minus SD plates containing 2% glucose or 2% galactose, where the expression of Ate1 is not induced or induced, respectively. Plates were incubated at 30 °C and images were taken after 3 days.

To test if the elevation of Ate1 would trigger the generation of mitochondrial ROS, we measured the level of mitochondrial ROS in yeast over-expressing Ate1 in different timepoints (up to 12 h, where the cell is expected to enter the apoptotic program [9]). However, we did not observe any significant increase in mitochondrial ROS levels throughout this time course (Fig. 9A). To test this from a different angle, we employed superoxide dismutase Sod1 or Sod2, which are known to neutralize or mitigate ROS toxicity [58, 59]. While Sod1 is mainly located in the cytosol and partially in mitochondrial IMS [60, 61], Sod2 is exclusively located in the mitochondrial matrix [61–63]. We found that, while the expression of Sod1 and Sod2, driven by the GPD promoter, were significantly increased in mRNA and protein levels (Fig. 9B, C), they generated no protecting effects in Ate1-induced cell death (Fig. 9D). Consistently, as mentioned earlier, the treatment of yeasts with quercetin, which is an inhibitor of mitochondrial ATP synthesis and also an anti-oxidant known to quench ROS in both mitochondria and cytosol, generates little effects on the Ate1-induced cell death (Fig. 6D). Therefore, while we previously showed that the presence of oxidative stress would upregulate Ate1 expression and would induce its translocation to mitochondria, the elevated Ate1 does not directly induced ROS production. Also, after Ate1 level is elevated, it does not require ROS to further carry on the cell death program.

### Cytosolic pathways, including UPS, autophagy, and UPR are not required for the execution of Ate1-induced cell death

Ate1 was traditionally considered as a cytosolic protein and its functions were extensively characterized in the context of promoting protein degradations in relation to UPS, autophagy, and UPR. As such, past studies relevant to Ate1 in cell death were mainly designed to test the effects of Ate1 on the cytosolic proteome. However, our above findings for the role of Ate1 in mitochondria-dependent pathway compelled us to re-examine these cytosolic pathways in the context of Ate1 and cell death.

**Fig. 10:**
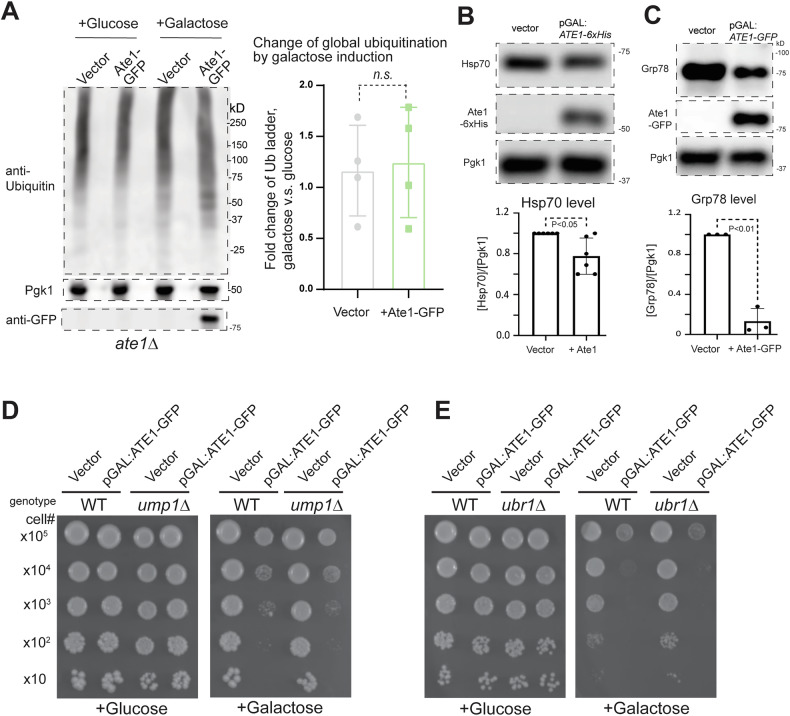
Ate1-overexpression does not lead to elevation of global ubiquitination and Ate1-induced cell death is not directly dependent on the functions of the ubiquitin-proteasome system. **A** Left panel displays representative Western blots depicting global ubiquitination levels in *ate1*Δ yeast with either pGAL:*ATE1-GFP* or empty vector, which were induced for 6 h with 2% galactose in liquid media. The level of Ate1-GFP was probed with anti-GFP. Pgk1 was used as a loading control. The right panel display quantification of the fold-change of total ubiquitin ladder signals by switching from glucose to galactose media (with raffinose media as a transition condition), which was normalized by Pgk1 loading. A *p* value > 0.05 is considered nonsignificant “*n.s*.” (*n* = 4). **B** Representative Western blot showing the expression levels of cytosolic stress response protein HSP70 in the yeast cells carrying the pYES2-pGAL:*ATE1-6* *×* *His-*URA3 expression vector or the empty control vector, which were induced with 2% galactose for 6 h in liquid media. The level of Ate1 was probed with antibody against 6 × His tag. The lower panel shows the densitometric analysis of the cytosolic Hsp70 levels as expressed in fold difference after normalization to the internal protein loading control Pgk1 (*n* = 6). **C** Similar to (**B**), except that the level of Grp78/HDEL, a maker of the endoplasmic reticulum unfolded protein stress response, was shown. The quantification was based on 3 independent repeats (*n* = 3). **D** Growth assay of WT or *ump1*Δ yeast carrying either pYES2-pGAL:*ATE1-GFP-*URA3 or empty vector. The growth was measured by a serial dilution growth assay on Ura-minus SD plates containing 2% glucose or 2% galactose, where the expression of Ate1 is non-induced or induced, respectively. Plates were incubated at 30 °C and images were taken after 3 days. **E** Similar to (**D**), except that *ubr1*Δ was used to compare to WT yeasts.

Ate1-mediated arginylation was long considered a flagging signal for the recognition of UBR family E3 Ubiquitin (Ub) ligases. As such, an elevation of Ate1 was well anticipated to cause excessive ubiquitination, which in turn could trigger PCD by at least two scenarios. First, the presence of excessive ubiquitinated proteins may clog proteasome and/or autophagy systems, which can then lead to unfolded protein stress and cause apoptosis and other forms of PCD. Second, an over-activation of arginylation may lead to inadvertent depletion of proteins that are otherwise essential for maintaining cellular viability. To test the first possibility, we examined the global ubiquitination levels because an elevation of ubiquitin ladder/smear would be expected if the degradation machinery is clogged. However, we found that over-expressing Ate1 did not lead to a significant increase of ubiquitin smear (Fig. 10A). Consistently, when we examined the level of cytosolic heat-shock protein 70 (Hsp70), a marker for cytosolic unfolded protein stress, we found it was not increased, but moderately decreased, when Ate1 is over-expressed (Fig. 10B). Similarly, when we examined the ER UPR stress, which can be reflected by the increased of Grp78, we found that the over-expression of Ate1 leads to a significant reduction of UPR (Fig. 10C). While the biological significance of these changes await to be clarified, they nevertheless suggest that Ate1 overexpression per se did not increase ER or cytosolic unfolded protein stress, making these factors unlikely to be the direct cause of cell death in this context. To test the second scenario, we used yeasts with the deletion of *UMP1*, which is expected to compromise proteasome activity and reduce protein turnover. However, this deletion does not provide any protective effects against Ate1-induced cell death (Fig. 10D). As a further validation, we used yeast with the deletion of the only UBR enzyme (Ubr1) in yeast, which is expected to block the ubiquitination of arginylated proteins, and again we saw no rescue effects by this knockout (Fig. 10E). Therefore, the cell death mediated by elevated Ate1 is not very likely to be mediated by an excessive degradation of cytosolic proteins.

Finally, while emerging evidence has suggested a linkage between protein arginylation and autophagy [21, 22, 28, 64–67], the role of autophagy in PCD is less straight forward. A normal level of autophagy is generally protective, but a disruption or an over-activation of autophagy can both lead to cell death [68]. However, as we already showed, the over-expression of Ate1 did not significantly affect autophagy in the tested timeframe (Fig. 5C). Also, the knockout of *ATG1*, an autophagy-essential gene, did not prevent Ate1-induced cell death in the tested condition (Fig. 5D).

## Discussion

Ate1 (and with its gene duplicate product Ate2 in plants) is the sole enzyme in most eukaryotes that catalyses arginylation, a PTM that has been compared to phosphorylation because they both add a charged and bulky chemical group to the target protein. While a substantial amount of data has unambiguously indicated the involvements of Ate1 or arginylation in cell death, the mechanism and even the exact role of Ate1/arginylation in such processes remain controversial due to conflicting reports. Our finding for the first time has pointed out that Ate1 by itself regulates PCD mainly by driving the intrinsic, mitochondria-dependent apoptotic pathway.

Mitochondria play a central role in many PCD-related scenarios. These include, but are not limited to cancer, embryogenesis, tissue development and regeneration, injuries, and aging. By demonstrating the role of Ate1 as a driver of the mitochondrial PCD pathway, our study provides important mechanistic insights for understanding the above-mentioned conditions, all of which are known to involve Ate1 or arginylation. Furthermore, our finding shows that Ate1 is involved in the activation of mPTP and MOMP processes, two of the most conserved mechanisms in PCD. This role of Ate1 is consistent with its nature as a product of gene transfer from α-proteobacteria, the ancestor of mitochondria. Given the extensive research on mPTP and MOMP, it is surprising that the involvements of Ate1 in these processes have remained largely overlooked. Such a gap of knowledge further highlights the nature of the studies of Ate1/arginylation as a nascent research direction. This also indicates that the mechanisms governing mitochondrial PCD is likely more complex than previously assumed.

In our study, we showed that the elevation of Ate1 lead to the activation of mPTP and MOMP, which is expected to initiate release of mitochondria-contained cell death factors such as Cyt-*c*, Aif, and EndoG. Consistently, we found that Ate1-induced cell death is suppressed by the deletion of the *AIF1*, one of the mitochondria-contained factors that can independently execute PCD. However, little or no effects were observed with the deletion of *NUC1* (yeast EndoG) or CYC3 (required for the maturation of Cyt-*c*). Similarly, the deletion of yeast metacaspase (*YCA1*), which is expected to respond to the release of Cyt-*c* and trigger the execution of apoptosis, does not prevent Ate1-mediated lethality. While it is still too early to make a definite conclusion at this stage, it is possible that Aif takes a more dominant role in the execution of yeast apoptotic program, while Cyt-*c*, caspase, or EndoG play redundant roles.

Ate1/arginylation is conventionally considered a key player in the ubiquitination and proteasome-dependent degradation of many proteins in the cytosol. Recent studies also suggest its role in promoting autophagy [21, 22, 28]. Our data demonstrated that, after Ate1 is elevated and enriched in the mitochondria, it is fully sufficient to execute the mitochondrial PCD process with little dependency on the cytosolic UPS, UPR, or autophagy machineries. Still, we need to carefully point out that, this study was not designed to rule out the involvements of cytosolic pathways. For instance, as shown in previous reports [5, 9, 69] and this study, the presence of acute stress is essential to trigger the increase of Ate1/arginylation in the cells. However, in this study (except for the data in Figs. 1 and 2), the elevation of Ate1 was mainly achieved by using recombinant expression without involving external stressors. Therefore, our data did not exclude potential contributions of UPS/UPR/authophagy in the elevation or relocation of Ate1 or the subsequential PCD process in the presence of external stress. For similar reasons, our data cannot rule out the potential impact of Ate1/arginylation in necrosis or macro-autophagy, particularly when external stress are involved. Further understanding of these processes will need to be obtained by additional investigations.

Our data also indicates several novel interactions that were previously overlooked and should be further clarified to obtain a better understanding of PCD. For example, our data suggest that the elevated Ate1 appears to suppress the cytosolic and ER unfolded protein stress signals, which by itself can also trigger PCD given the right context. It is possible that Ate1 and UPR constitute negative feedback or a competing relationship for the regulation of PCD. While still speculative, this effect may be explained by the known impact of arginylation on the turnover of some of the pro-apoptotic proteins or protein fragments associated with UPR. These include calreticulin and Grp78, which may be retro-transported back to cytosol from ER during the crisis of UPR.

Our new findings also introduce new questions that were previously non-existent. Particularly, we are curious how Ate1 regulates mPTP and MOMP processes. Our current data appear to indicate that these are mainly controlled by the intra-mitochondrial Ate1. However, this question cannot be easily addressed right now because most existing studies about Ate1 were focused on the cytosol or nucleus. While a couple of mitochondrial proteins were sporadically identified in past screenings for arginylation substrates [70, 71], most of them have no obvious relevancies to cell death. Further convoluting is that those studies were not even designed to include mitochondrial fraction in the first place. As such, it would be premature to speculate how Ate1 trigger the mitochondrial cell death pathway. We are excited to see how future investigations will further clarify these open questions, and contextualize our understanding of this ancient protein’s involvement in mitochondrial regulation and eukaryotic cell death.

## Materials and methods

### Yeast strains

The *S. cerevisiae* strains, wild-type or knockout, are mainly based on the parental strain BY4741 (*MATa his3Δ1 leu2Δ0 met15Δ0 ura3Δ0*) unless otherwise indicated. In several experiments where knockouts are not available for the BY4741 stain, the W303-1A strain background (*MATa leu2-3,112 trp1-1 can1-100 ura3-1 ade2-1 his3-11,15 ybp1-1*) was used. These yeasts were obtained from Horizon Discovery. The identity of each knockout strain was confirmed with PCR genotyping. The strain containing a GFP-fused to the endogenous Ate1 was based on the BY4741 strain and was originally obtained from Open Biosystems (now part of Horizon Discovery) as described in our previous work [9].

### Culture of yeast

The below yeast culture media were used:

YPD media: 2% glucose, 1% yeast extract, 2% peptone.

SD (Synthetic Defined) Media (per 1000 ml): Yeast Nitrogen Base, 1.7 g, Ammonium sulphate, 5 g, Dextrose/galactose/raffinose, 20 g, required amino acids, 50 mg, uracil (if required), 50 mg.

To prepare solid media plates, 2% agar was added to the liquid media before autoclave.

The yeasts were incubated at 30 °C to grow, unless otherwise indicated.

For the serial dilution growth assays, a single colony of yeast was inoculated in liquid medium and allowed to grow to the log phase. The spot platting of serial dilutions was performed as described before [72].

### Yeast cell transformation

Mid-log phase yeast cells with optical density (O.D.) at 600 nm corresponding to 0.6 was grown in YPD liquid media and harvested. Yeast cells were washed twice with sterile water and added with transformation reaction mix containing polyethylene glycol (PEG 3000), lithium acetate, carrier DNA (salmon sperm DNA) and desired plasmid. Cells were vortexed completely and subjected to heat shock at 42 °C for 15 min as described earlier [9]. Transformants were selected on SD agar plate lacking essential supplements such as either uracil or leucine or tryptophan based on the experimental requirements.

### Molecular cloning and preparation of plasmids

High-fidelity DNA polymerases such as Herculase or Pfu turbo (from Agilent) were used to perform the DNA amplification. Restriction digestion and ligation were performed with corresponding restriction enzymes and T4-DNA ligase (NEB catalogue number M0202S) from New England Biolab. Chemical competent *Escherichia coli* TOP10 (Thermo-Fisher Scientific) were used for plasmid transformation.

PCR reactions were performed in a Veriti Thermo Cycler (Applied Biosystems) or a T100 Thermo Cycler (BioRad). Most primers were ordered from Sigma-Aldrich.

The vector with the “pGAL” promoter in this manuscript is referred to the PYES2 plasmid, which contains the galactose driven promoter. It was obtained from (Life Technologies, # V825-20).

The cloning of DD-β15-mCherryFP, arginylation reporter: the cDNA of arginylation reporter contains an N-terminal ubiquitin followed with a stretch of 15 amino acid from the N-terminus of beta actin (DDIAALVVDNGSGMC) [73], a linker region, and then the coding region for C-terminal mCherryFP. It was modified from a similar reporter DD-β15-GFP as described in our previous work [9]. The construct is cloned in a yeast expression vector, pGPD2 (Addgene # 43972), at EcoR1 and Xho1 sites. The following primers were used for this cloning: 5′–ATGTGCGGGATCCACCG–3′ and 5′-ACCTCGAGTTAATGGTGATGGT -3′.

The cloning of pGAL-Ate1-GFP-Ras2: It is modified from the original pGAL-Ate1-GFP, which was hosted at the pYES2 yeast expression backbone vector as described in our past work [9]. The sequence for the last 37 amino acids of Ras2 was amplified from the Ras2 gene contained in a pYES2 plasmid; its C-terminus was amplified using a Ras2-specific forward (ATATGCATGCAATAGTAAGGCCGGTCAAG) primer containing an SphI cut site, and a plasmid-specific reverse primer (CGTACACGCGTCTGTAC). The amplified PCR product was extracted and digested with SphI and SalI before being ligated into a similarly digested Ate1-GFP-containing plasmid.

The inducible BAX-expression vector (pBM272-GAL-BAX-URA), with mouse BAX gene under Gal promoter and a URA3 selection marker, was acquired from Addgene (Plasmid #8770)

The constitutively expression vector for Bcl-xL (pBF339-ADH-BCLxL-TRP), with mammalian Bcl-XL gene under ADH1 promoter and a TRP1 selection marker, was acquired from Addgene (Plasmid #8773).

The constitutively expression vector for SOD1 and SOD2 are pGPD2-LEU2, which contains a GPD promoter and a LEU2 selection marker.

The coding sequence of yeast NHP6A-eGFP was obtained from Dr. Reid C. Johnson (at the University of California, Los Angeles). It was originally contained in a shuttle vector pRJ1522 [44]. It was subcloned into a pESC-LEU2 vector, which contains a LEU2 selection marker, using NotI/XhoI restriction sites.

The construct (pYES2-PGAL1-Mt_Cyb2delta-Ate1-GFP or PYES2-PGAL1-IMS_Cyb2-Ate1-GFP) expressing mitochondrion-localized (matrix) or Intermembrane space-localized Ate1 was modified from the parental Ate1-GFP yeast expression plasmid was described in refs. [9] [74]. A three-part ligation strategy was used. Fragment 1 was generated by double digestion of pYES2 vector with Age1 and Xba1 restriction enzyme. Fragment 2 harbouring either the IMS-targeting sequence the matrix-targeting sequence of *S. cerevisiae* Cytochrome b2 (Cyb2) was PCR amplified from pYX233-b2delta-DHFR (addgene plasmid #163760) or pYX233-b2-DHFR (addgene plasmid # 163759) using the forward primer GTCCTCGTCTTCACCGGTCGCGTTCCTGA and the reverse primer GGGGTACCTTGAAGGGGACCCAATTTTTTCTCGGG. The full-length Cyb2 tag will lead the fusion protein retained in the IMS. After the cleavage by an IMS-specific protease, an extra peptide of 81 Aa is expected to remain on the fusion protein [40]. In b2delta, the stop-transfer signal (transmembrane domain) of cytochrome b2 was deleted (19 amino acids) so that the protein with this tag is targeted to the mitochondrial matrix [41]. After the cleavage by a matrix-specific protease, an extra peptide of 116 Aa is expected to remain on the fusion protein. Fragment 2 was restriction-digested using Age1 and Kpn1 enzymes. Fragment 3 containing Ate1-GFP was restriction digested, and gel-purified from pYES2-PGAL1-Ate1-GFP vector and subjected to three-part ligation using the T4 DNA ligase (NEB catalogue number M0202S).

### RNA extraction, cDNA preparation, and Quantitative PCR

Yeast transformants were grown in appropriate selection media for 12–14 h. Next day, the cultures were reinoculated at an optical density (OD_600_) of 0.2 and allowed to grow until an OD_600_ of 0.6. To induce the expression of the ATE1 gene, which is under the control of a galactose-inducible promoter, the cultures were subsequently incubated overnight in galactose-containing media. Following induction, total RNA was isolated from the yeast cells using the Quick mRNA kit (Qiagen, Cat. No. 74104) according to the manufacturer’s instructions. The corresponding cDNA was synthesized using an iScript TM cDNA synthesis kit from BIO-RAD (Cat No. 1708891), following instructions given by the manufacturer.

The quantitative PCR (qPCR) was performed using Takara TB Green kit with Premix Ex Taq (cat #RR820A) in the CFX96™ Real-Time PCR Detection System coupled with the C1000™ Thermal Cycler (BIO-RAD). The primers and their expected product size for each gene are:

For SOD1 gene: SOD1-qF (GTCTCTGCTGGTCCTCACTT) and SOD1-qR (GTGGATAACGACGCTTCTGC), with 189 bp expected product;

For SOD2 gene: SOD2-qF(GCTGGACGTTGTTCAAACCT) and SOD2-qR (TCAGATCTTGCCAGCATCGA) with 190 bp expected product:

For ACT1 gene: ACT1-qF (TGTCACCAACTGGGACGATA) and ACT1-qR (GGCTTGGATGGAAACGTAGA) with 190 bp expected product.

### Induction of Ate1 expression in yeast cells in liquid media

The yeasts (carrying Ate1 expression vectors with URA3 selection markers, or the empty vector control) were grown in SC -Ura liquid media supplemented with 2% raffinose until O.D. 600 nm reach 0.5–0.6. Unless otherwise indicated, the expression of Ate1 was achieved by adding 2% galactose at 30 °C for 5–6 h, which is not expected to trigger major (more than 50%) cell death events by the overexpression itself [9].

To induce oxidative stress, cells were treated with 5% NaN_3_ for 30 min at 30 °C.

### Chemical treatments of yeast cells on solid media plate

Yeast plates were made as previously described. To make the various inhibitors containing plates, a working stock was created using suitable solvent (DMSO for oligomycin/quercetin; ddH_2_0 for alpha-ketogluterate). After autoclaving the agar-containing media, it was allowed to cool till it was tolerable to touch (below 50 °C). At this point the required amount of the chemical was added (from the working stock) into the media and allowed to stir till solution was homogeneous. Plates were then poured using this homogenous solution, and allowed to solidify for one day before use in experiment.

### Preparation of whole-cell lysates and mitochondrial fraction

To prepare whole-cell lysates, the yeast cells were lyzed by vortexing with glass beads in a 2× Laemmli SDS-loading buffer (for direct analysis). Alternatively, total protein extracts were prepared using sodium hydroxide and trichloroacetic acid as described previously [75].

To prepare mitochondrial fraction, the yeast cells were harvested by centrifugation. The mitochondria were isolated as per the protocol described by the manufacturer (abcam#ab178779) with following modifications. Briefly, yeast cells were harvested and rinsed with water. Cell pellets were either stored at −80 °C or processed immediately. Cells were suspended in Buffer-A containing 1 mM DDT and incubated at 30 °C with gentle shaking for 10 min. Cells were pelleted and resuspended with buffer-B containing lyticase. Cell suspension was incubated at 30 °C for 30–45 min. The successful formation of spheroplast was validated under optical microscope. After centrifugation at 600 × *g* for 5 min a lysis buffer containing protease inhibitor cocktail was added to the pellet and resuspended completely. Cell lysis was carried out using Dounce homogenizer 1 × 15 strokes on ice. Unlysed cell debris were pelleted by centrifugation at 800 × *g* for 5 min twice. The supernatant containing mitochondria was pelleted by centrifugation at 12,000 × *g* for 10 min. To remove contaminated cytosolic and nuclear proteins the resuspended mitochondrion fraction was treated with proteinase K at 5 µg/ml concentration for 45 min on ice. The reaction was stopped by adding 20% TCA. Proteins were pelleted by centrifugation at 18,000 × *g* for 15 min at 4 °C. The protein pellet was washed once with ice-cold 100% acetone and centrifuged at 18,000 × *g*.

For the cytochrome c-related fractionation, the following procedure was followed: Yeast cells were induced with galactose, harvested, and washed once with 1× PBS. Yeast cells were then resuspended in 650 mM DTT and 250 mM EDTA and incubated for 10 min at 30 °C and 75-rpm gentle shaking. Cells were then spun down, supernatant removed, and washed with Sorbitol Buffer (1 M sorbitol, 0.15 K2HPO4, pH 7.4) once. Afterwards, the net weight of the yeast pellet was taken and yeast were resuspended in a mixture of sorbitol buffer (7 mL/g wet weight of pellet) and Zymolase (3 mg/g wet weight of pellet) and shaken at 50 rpm for 35 min at 30 °C. After digestion with Zymolase, the resulting spheroplasts were pelleted at 3000 × *g* and washed gently with sorbitol buffer. Pellets were then placed into a pre-chilled douncer homogenizer on ice and dounced a total of 10 times. A final volume of 100 µL of homogenate was taken (and if needed, sorbitol buffer was added) and spun down at 5000 × *g* for 10 min at 4 °C on a desktop centrifuge. Supernatant was isolated and placed in a separate tube on ice. This isolated supernatant was the centrifuged at 15000 × *g* for 20 min at 4 °C. The resulting supernatant was isolated from this and collected as the “cytosolic” fraction, while the residual pellet was considered the “mitochondrial” fraction. 2× Laemelli buffer was added to each fraction to each an equal final volume, and samples were boiled for 10 min before use in SDS-PAGE analysis.

### Analysis with SDS-PAGE and Western blot

All protein samples were prepared in Laemmli SDS-loading buffer and heated in a boiling water bath for 10 min as described [73]. The samples were separated by electrophoresis on SDS-PAGE. Unless otherwise indicated, 4–20% Mini-PROTEAN® TGX™ Precast Protein Gels from Bio-Rad were used.

The freshly separated bands were transferred to nitrocellulose membrane for western blot analysis.

Western blot blocking reagent was obtained from Roche (catalogue number 75255200)

The primary antibodies include:

monoclonal mouse anti-GFP (from Roche, clone 7.1 and 13.1, Cat# 11814460001)

Rabbit anti-yeast alpha tubulin (Abcam EPR13799)

anti-yeast- phosphoglycerate kinase1 (Pgk1) (Thermofischer scientific # 459250),

Rabbit anti-yeast Cmc2 was a gift from Dr. Antonio Barrientos (University of Miami)

mouse anti-Grp78 (SCBT# HDEL Antibody (2E7): sc-53472)

HSP70 Monoclonal antibody (Proteintech catalogue# 66183-1)

The custom-produced rabbit anti-RDD antibody was ordered from Genscript INC as described in our previous work [9].

The rabbit anti-SOD1 antibody was a gift from obtained from Giovanni Manfredi (Weill Medical College of Cornell University) [76].

The rabbit anti-SOD2 antibody was obtained from Sigma-Aldrich (Catalog # HPA001814).

The secondary antibodies include:

Goat anti-Mouse IgG (H + L) Secondary Antibody, HRP (Invitrogen catalogue# 314303)

Goat anti-Rabbit IgG (H + L) Secondary Antibody, HRP (Invitrogen catalogue# 656120

To visualize the antibody signal, the following chemiluminescent kits were used:

Pierce^TM^ ECL western catalogue # 32106

SuperSignal™ West Femto Maximum Sensitivity Substrate catalogue # 34096

The chemiluminescent signals were documented by a GE Amersham Imager model 600 and analysed with an ImageQuant TL software pack (v8.1) and its 1D gel analysis module.

### Microscopy

Optical and fluorescent imaging of the cells were done using a Zeiss Observer microscope, which was equipped with a series of objectives and the Zen-Pro analysis software.

### Flow cytometry (FACS)

FACS analysis was performed on a BD Canto-II flow cytometer, which is hosted by the flow cytometry core facility of the Sylvester Comprehensive Cancer Center at the University of Miami.

### Evaluation of yeast cell viability under Ate1 overexpression by serial dilution assay

The yeast cells were transformed with the overexpression vectors carrying different constructs of *ATE1*, in comparison of the empty vector pYES2, all of which contain the URA3 selection gene markers. The yeast cells were grown to mid-log phase in a synthetically defined media lacking uracil (Sunrise Science Products; Catalog #: 1306-030). Optical density of yeast cells at 600 nm corresponding to 0.5 was harvested and thoroughly washed with sterile water. Cells were serially diluted and spotted on to SD-URA plates containing 2% of glucose, raffinose, or galactose, unless otherwise indicated. Plates were incubated at 30 °C for 2–5 days and photographed.

### TUNEL assay for apoptosis in yeasts

Yeast cells were first grown in raffinose-containing media for 20 h before being washed twice and resuspended in liquid dropout media containing 2% galactose (SDGal -Ura) and placed into an erlynmer flask (culture volume < 10% flask volume). Flasks were then placed into a shaking incubator (rpm 220) and incubated at 30 °C for 20 h. After 1 O.D. worth of cells were harvested and washed once with galactose-containing dropout media and resuspended in 1 mL of this same media, in an Eppendorf tube. Formaldehyde was added to final concentration of 3.7% (v/v), and the tubes were mixed by inversion several times before being allowed to sit at room temperature for 30 min. Cells were then washed three times with PBS, before being digested with Zymolase 100 T (24 µg/mL final; in PBS) at 37 °C for 60 min. Resulting spheroplasts were gently washed once with PBS before being applied to a microscope slide, and allowed to dry out (40 min at 37 °C). The rest of the procedure followed manufacturers instruction for “tissue section staining” using the Invitrogen Click-iT Plus TUNEL Assay.

### Nuclear staining with 4’,6-diamidino-2-phenylindole (DAPI) for yeasts

BY4741 yeast wild-type strain was grown in SD glucose complete media to mid-log phase at 30 °C. OD 600 nm corresponding to 1.0 was transferred to 1.5 ml eppendorf tube and add with 20 µl of DAPI stain stock of 0.5 mg/ml and incubate in dark with shaking at 120 rpm for 40 min in 30 °C incubator. Cells were pelleted by centrifugation at 3000 × *g* for 3 min, supernatant was discarded, and the cells were washed with 1 ml of 1 × PBS twice. DAPI stained cells were resuspended in 450 µl of 1 × PBS and incubated in ice for 5 min and fixed by the addition of 50 µl of 37% formaldehyde for 30 min in ice. Cells were centrifuged at 2500 × *g* for 3 min and washed twice with 1 × PBS and finally dissolved in 70 µl of 1 × PBS. Approximately 5 µl of the samples were placed on microscopic slides and images were taken.

### Propidium iodide (PI) staining for yeasts

BY4741 yeast wild type strain carrying pYES2 empty vector plasmid or pYES2 vector containing *ATE1*-6 × His was grown in selection media lacking uracil either in 2% Raffinose or 2% Galactose as a carbon source. OD 600 nm corresponding to 2.0 was harvested and washed once with sorbitol buffer (1.2 M sorbitol, 0.5 mM mgcl2 and 35 mM potassium phosphate buffer (pH6.8). Cells were treated with 7.25 units of zymolaseT for spheroplast formation in sorbitol buffer at 30 °C for 20 min. Cells were pelleted at 2500 × *g* for 3 min and washed twice with 1× binding buffer containing 10 mM HEPES (pH7.4) add PI staining solution at a final concentration of 0.5 μg/ml and incubate in dark at room temperature for 30 min. Cells were pelleted by centrifugation at 3000 × *g* for 3 min, supernatant was discarded, and the cells were washed with 1 × PBS twice. PI-stained cells were resuspended in 1 × PBS and incubated in ice for 5 min and fixed by the addition of formaldehyde (final concentration 3.7%) for 30 min in ice. Cells were centrifuged at 2500 × *g* for 3 min and washed twice with 1 × PBS and finally dissolved in 1 × PBS, before being placed on microscopy slides for image acquisition.

### Annexin V staining for yeasts

Wild-type BY4741 yeast cells harbouring pYES2 empty vector or pYES2 vector containing *ATE1*-6 × His was grown and spheroplast was prepared as described previously in the PI staining protocol. Wild-type cells treated with 10 mM H_2_0_2_ for 200 min were used as a positive control. After lyticase treatment cells were stained with Annexin V using FITC Annexin V Apoptosis detection kit (from BD Biosciences, Cat# 556547). Briefly, after spheroplast formation cells were washed twice with 1× annexinv binding buffer containing 1.2 M sorbitol and stained using 5 µl of Annexin V stock buffer in 150 µl reaction volume at room temperature for 30 min in dark condition. After staining, cells were washed twice with 1× PBS and finally resuspended in 70 µl of 1 × PBS. 5 µl of the samples were used for microscopic analysis.

### Image processing

The images obtained from Western blot or microscopy were processed in Adobe Photoshop 2025. Regions of interest were taken by cropping. The images may also be subjected to rotation, flipping, scaling, and the change of display intensity by adjusting the “Levels” while keeping the gamma value as “1.00”, which is considered a linear adjustment for brightness and contrast.

Individual images were assembled in Adobe Illustrator 2025 for publication figures.

The original Western blot images used in the figures of this study are displayed in full lengths in the Supplemental Fig. S2.

### Statistics

The tests performed in this study are biochemical or cell based. The samples size was determined based on our previous experience on similar study. Unless otherwise indicated, statistical significance was derived using unpaired two-tailed student’s test with error bars representing standard deviation. A minimum of *p* value at 0.05 is considered significant. For certain dataset, including those generated by the analysis of microscopy images where the values involve manual assignment by the operators, a test of the normal distribution is performed before the *t*-test, as indicated in the corresponding figure legends.

## Supplementary information

Supplemental Figure S1

Supplemental Figure S2

## Data Availability

Most data generated or analysed during this study are included in this published article and its supplementary information files. Additional datasets generated during and/or analysed during the current study are available from the corresponding author on reasonable request.
