# Supplementary material for: Arginyltransferase1 drives a mitochondria-dependent program to induce cell death
Source: Cell Death Dis. 2025 Aug 16;16(1):622. doi: 10.1038/s41419-025-07917-1 (PMC12357888; doi:10.1038/s41419-025-07917-1)
Supplement: Supplementary file 1 — Supplemental Figure S1 [file 41419_2025_7917_MOESM1_ESM.pdf]

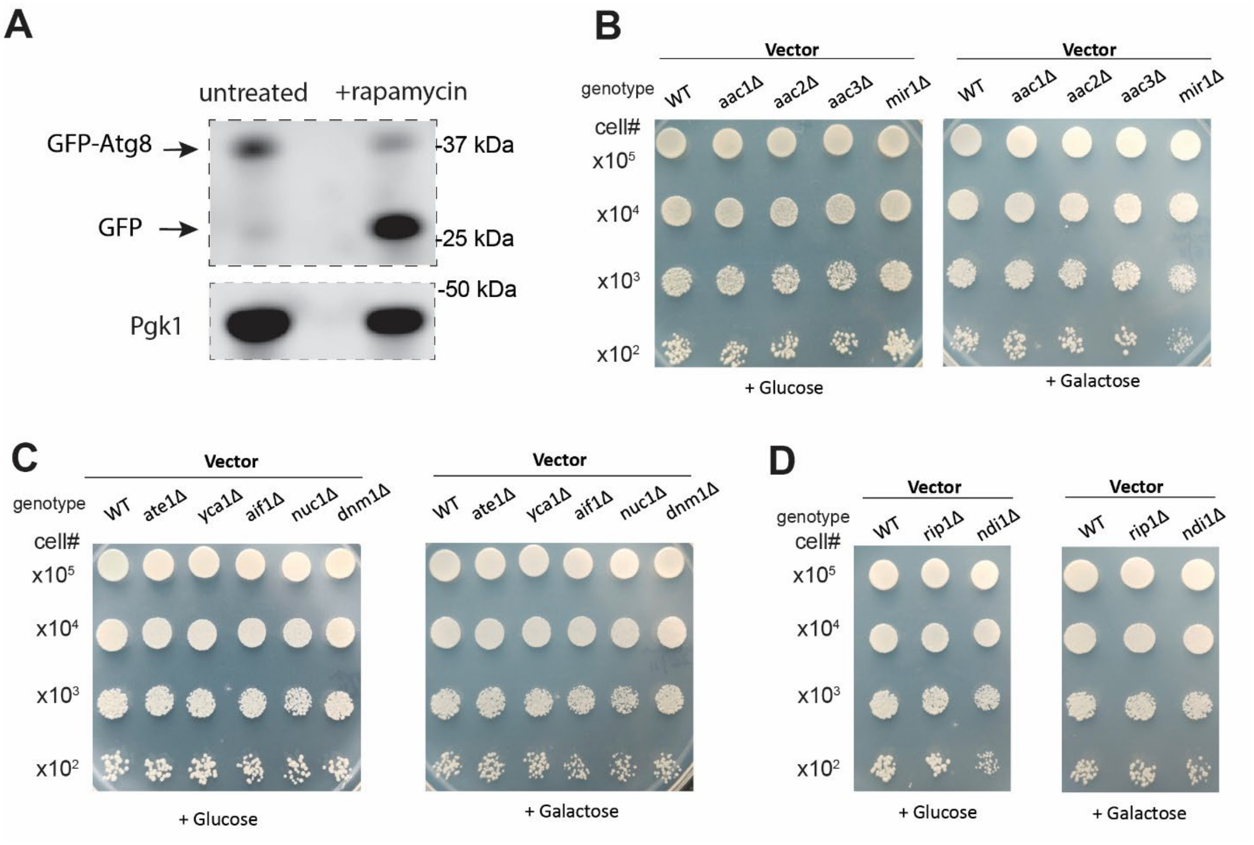

Supplemental Figure S1

A) Representative WB images of wild-type yeast cells (BY4741) carrying the autophagic reporter GFP-Atg8. The cells were either untreated or treated with 500nM rapamycin for 16 hours, which is expected to induce significant autophagy. The levels of the full-length GFP-ATG8 and the cleavage product (GFP) were probed by anti-GFP while Pgk1 was used as a loading control.

B) Serial dilution assays showing the growth in plates containing either 2% glucose or 2% galactose for these W303 yeast strains: WT, *aac1*Δ, *aac2*Δ¸ *aac3*Δ, and *mir1*Δ, all of which carry an empty PYES2-URA3 vector.

C) Serial dilution assays showing the growth in plates containing either 2% glucose or 2% galactose for these BY4741 yeast strains: WT, *aif1*Δ, *ate1*Δ¸ *yca1*Δ, *nuc1*Δ, and *dnm11*Δ, all of which carry an empty PYES2-URA3 vector..

D) Serial dilution assays showing the growth in plates containing either 2% glucose or 2% galactose for these BY4741 yeast strains: WT, *rip1*Δ, *ndi1*Δ, all of which carry an empty PYES2-URA3 vector.
