# Supplementary material for: Arginyltransferase1 drives a mitochondria-dependent program to induce cell death
Source: Cell Death Dis. 2025 Aug 16;16(1):622. doi: 10.1038/s41419-025-07917-1 (PMC12357888; doi:10.1038/s41419-025-07917-1)
Supplement: Supplementary file 2 — Supplemental Figure S2 [file 41419_2025_7917_MOESM2_ESM.pdf]

These are the full-length images for those used in the figures in this manuscript. Please note that these images may be subjected to the adjustment of display level or image rotation.

Only the lanes containing proteins bands displayed in this publication were included.

Related to Figure 2 A

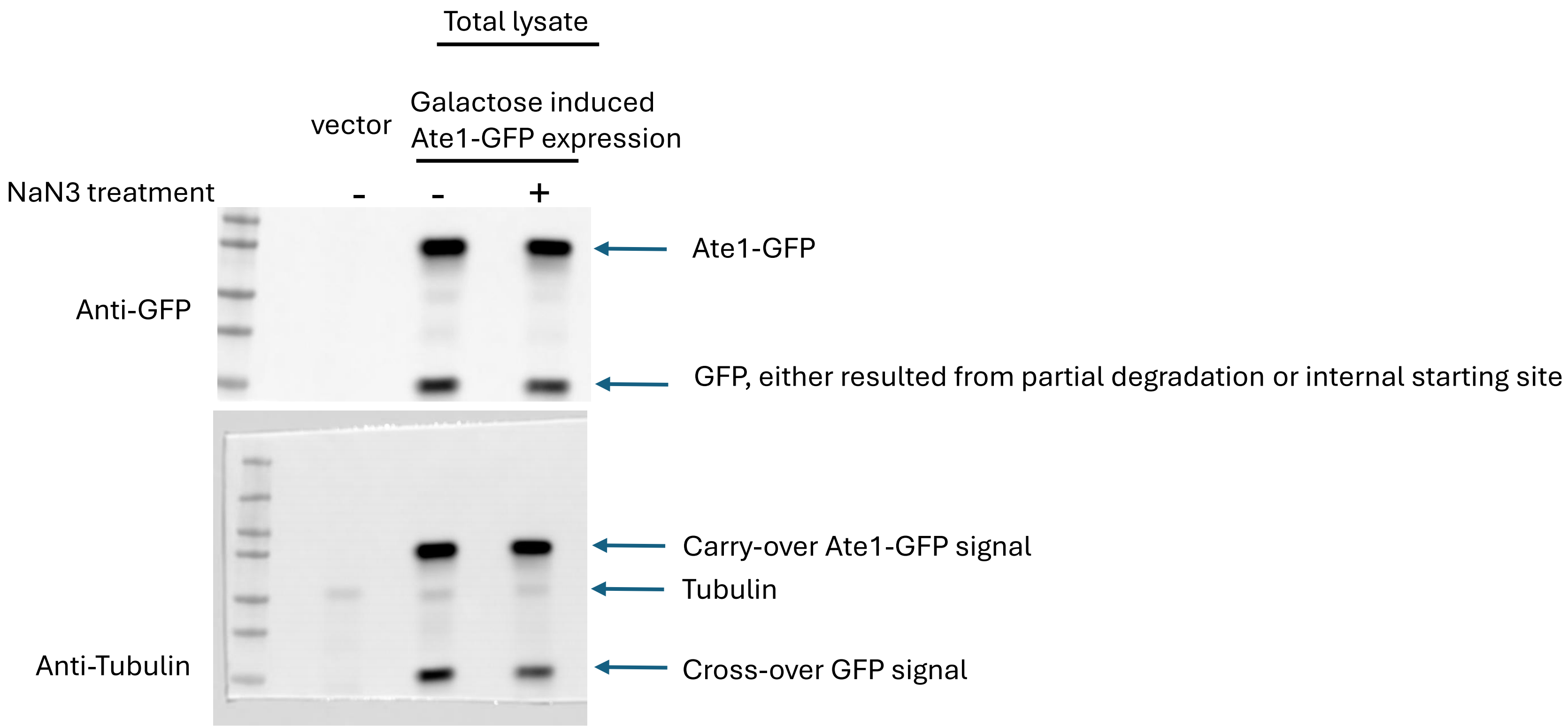

The membrane was first probed with anti-GFP and then anti-tubulin, leading to carry-over of signals.

Related to Figure 2 B

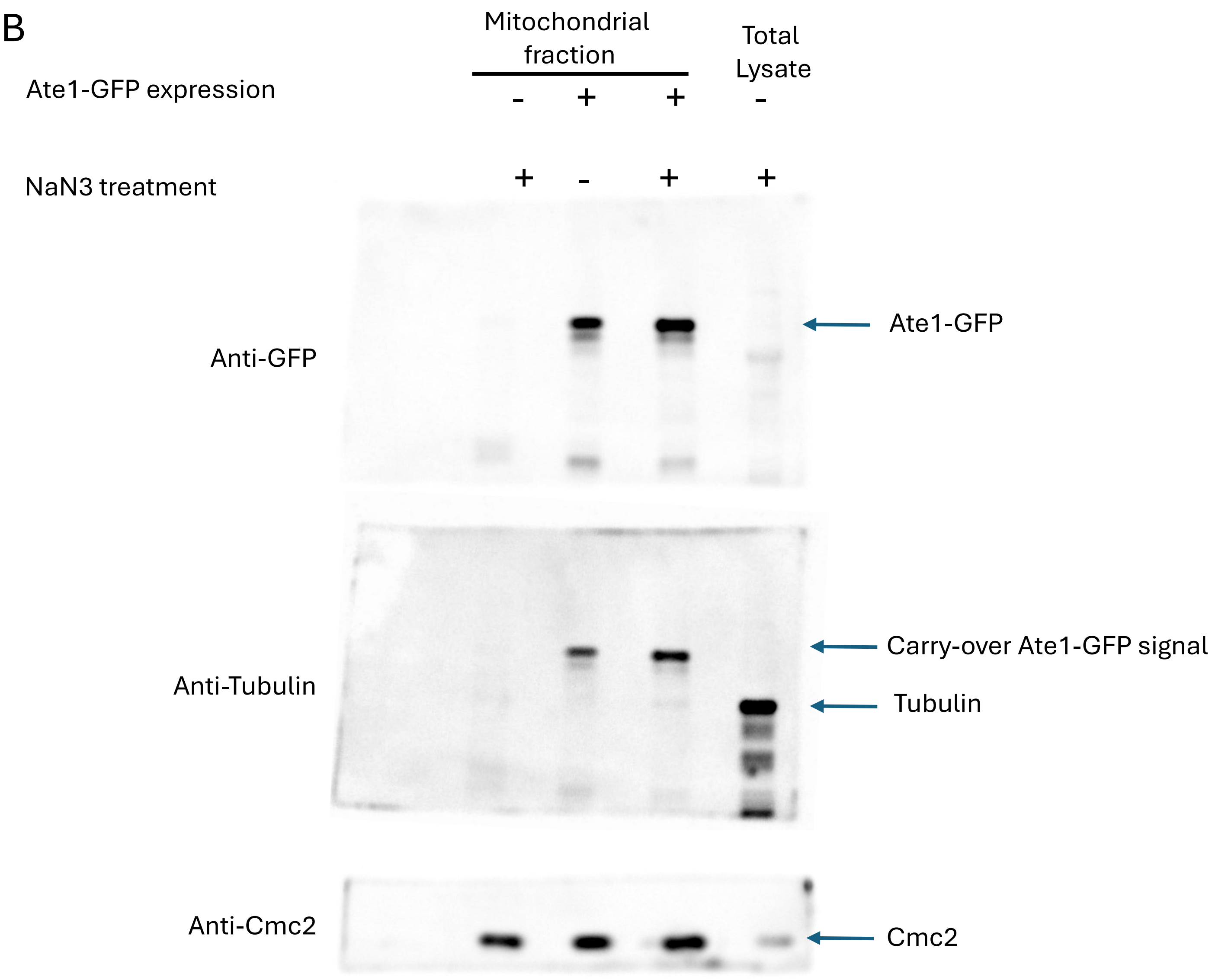

Note: The membrane was cut horizontally before incubating with the primary ani-Cmc2 and anti-GFP antibodies. The top membrane was first probed with anti-GFP and then anti-tubulin, leading to carry-over of signals.

Related to Fig 3C

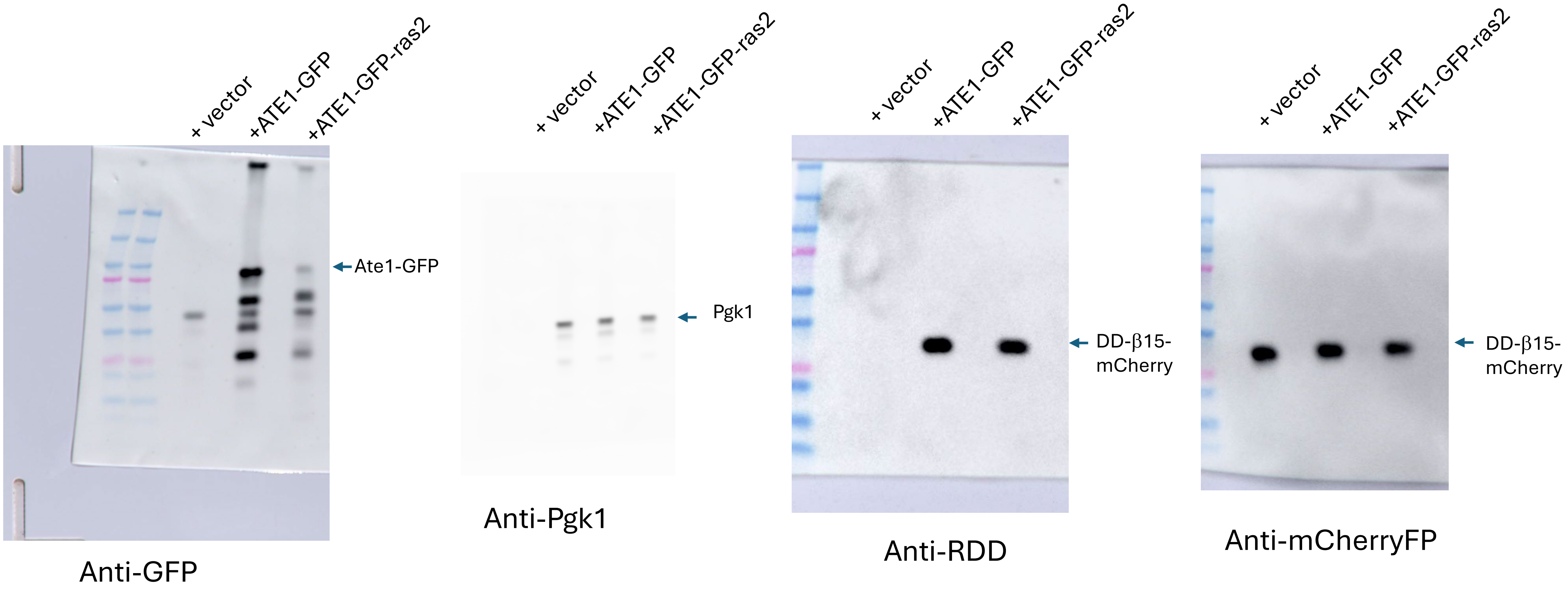

These two are from the same membrane, which was stripped and washed before the application of the new antibody

These two are from different membranes with the same loading to avoid carry-over of signals between different antibody recognizing the same protein

Related to Figure 3E

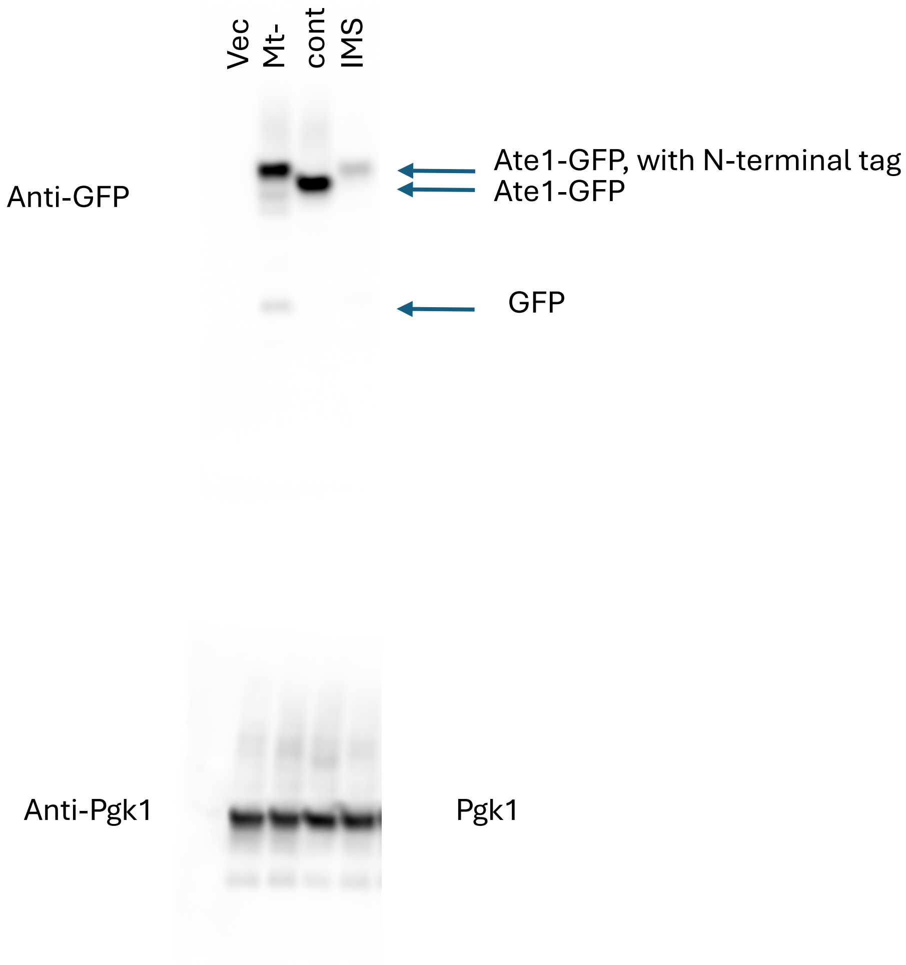

Related to Figure 3 F

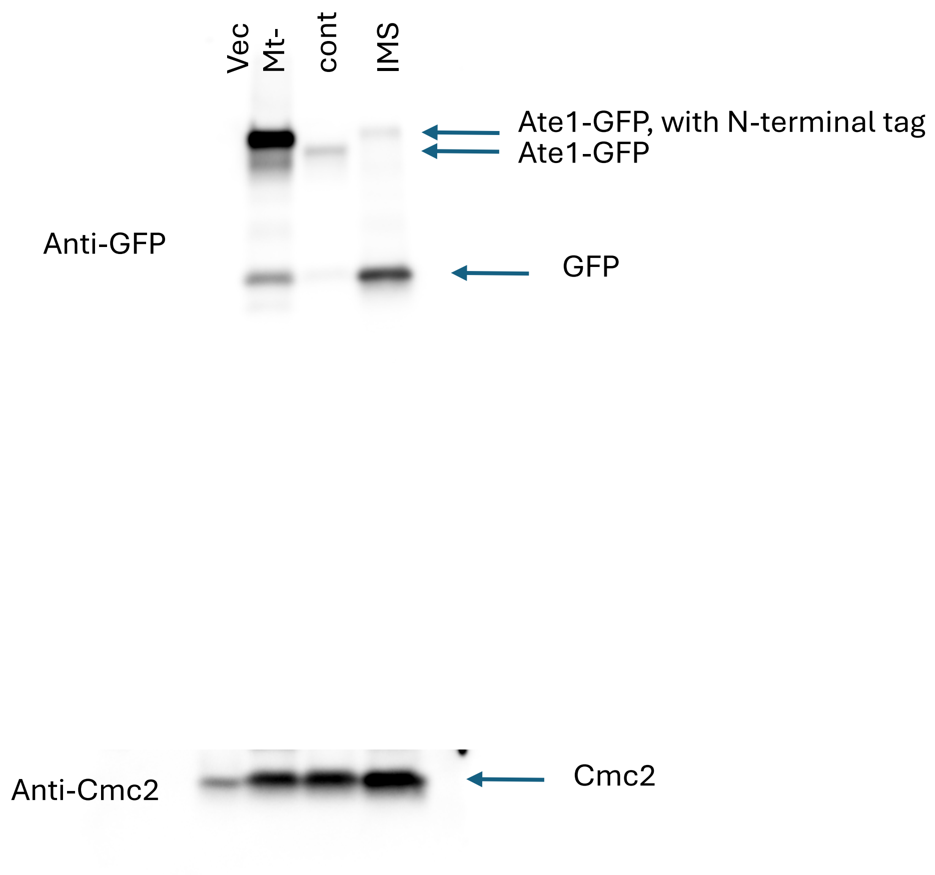

Note: The membrane was cut horizontally for separate probing by ani-Cmc2 and anti-GFP antibodies.

Related to Figure 5C

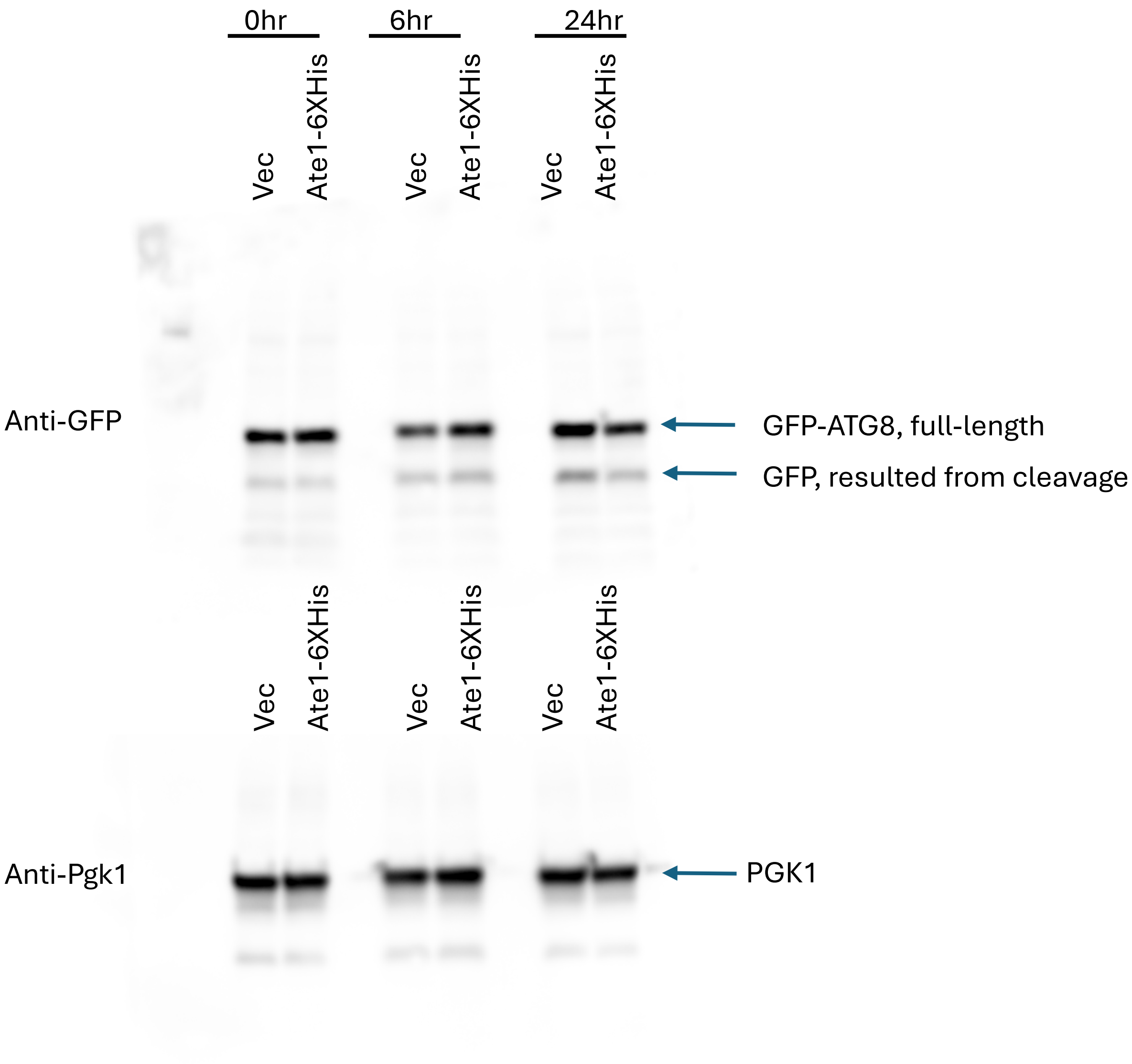

Related to Fig 7b

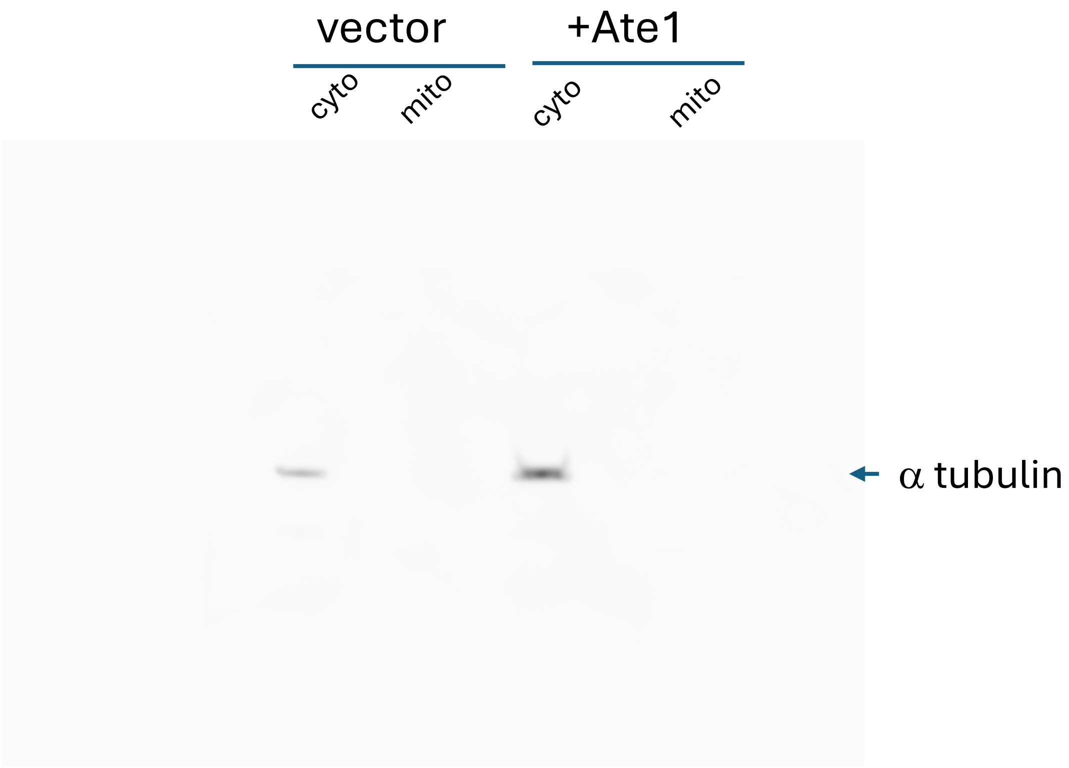

Anti-Alpha tubulin

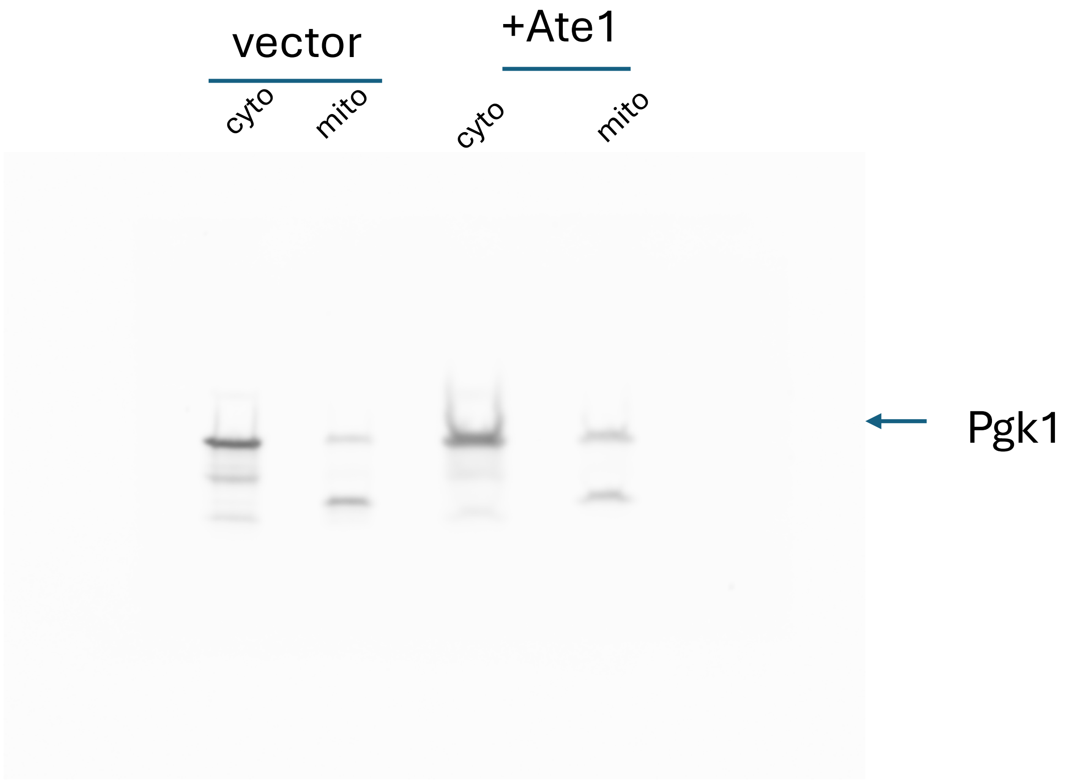

Anti-Pgk1

Related to Fig 7b

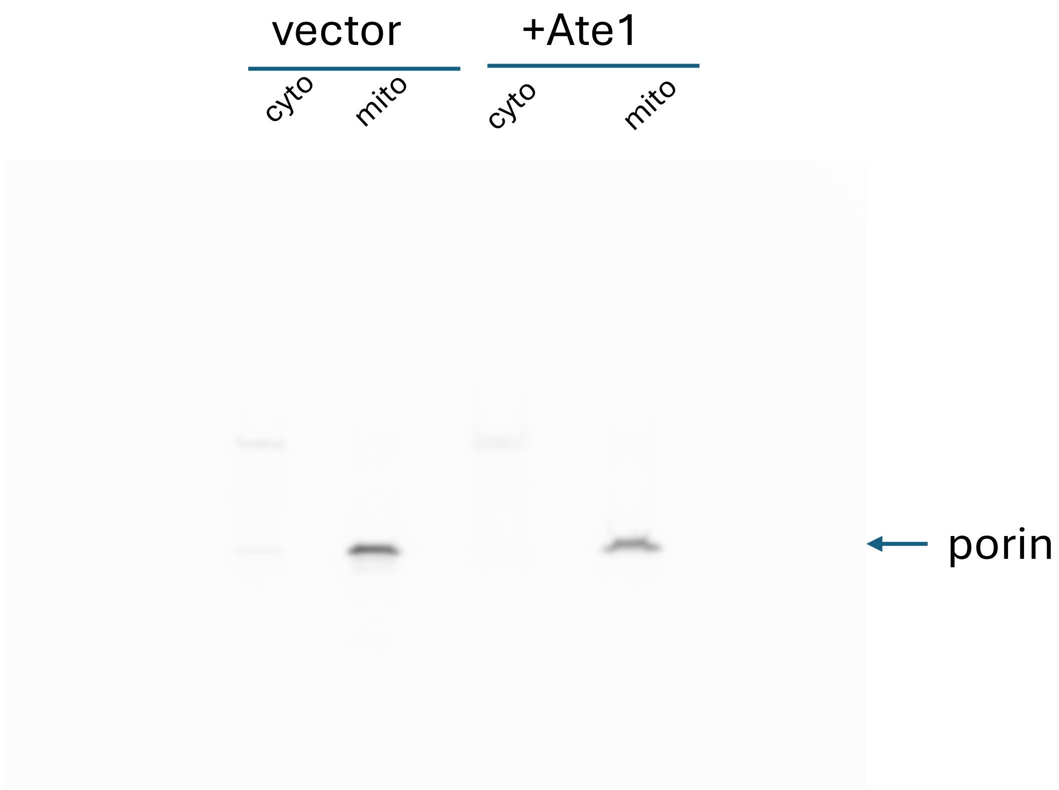

Anti porin

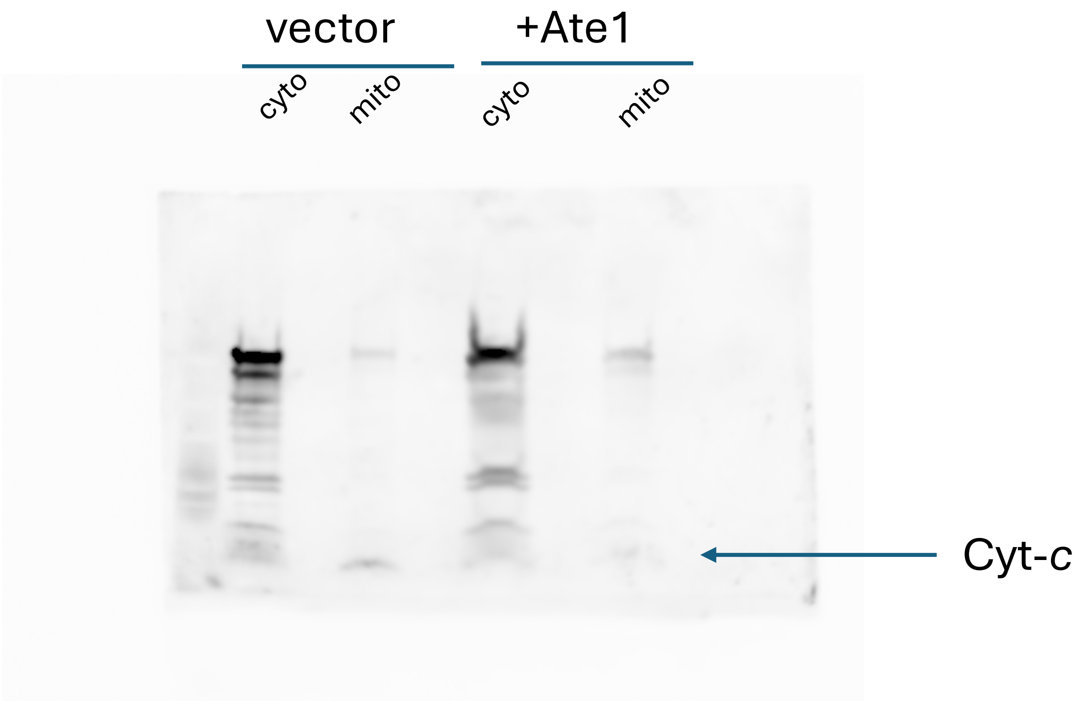

Anti cytochrome c

Related to Fig 9C

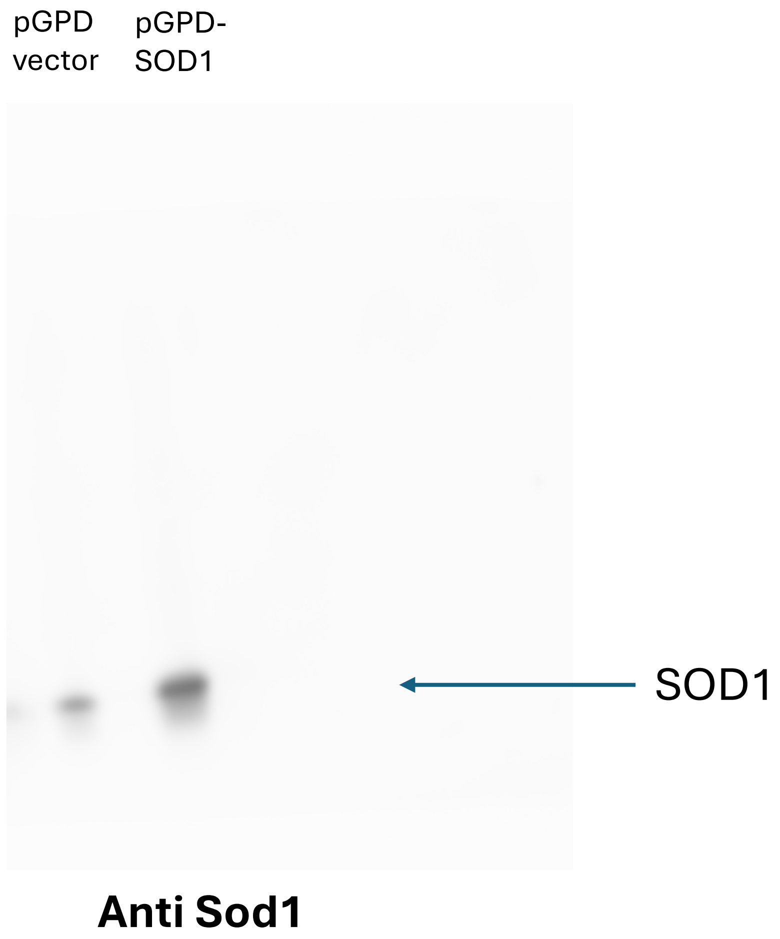

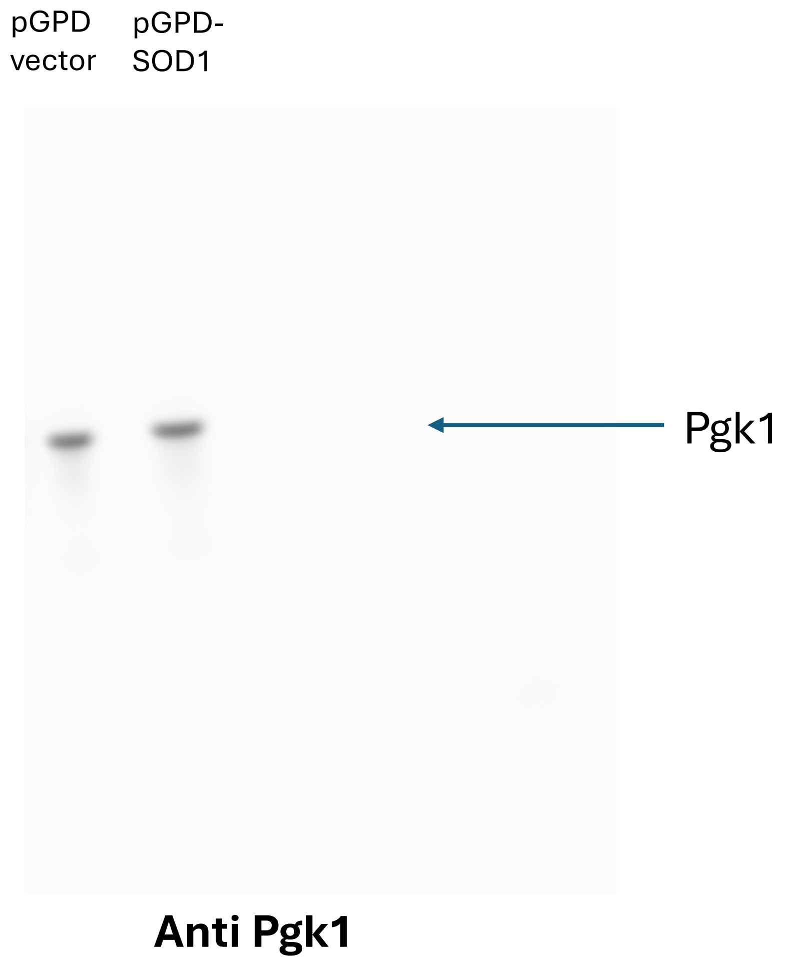

Related to Fig 9C

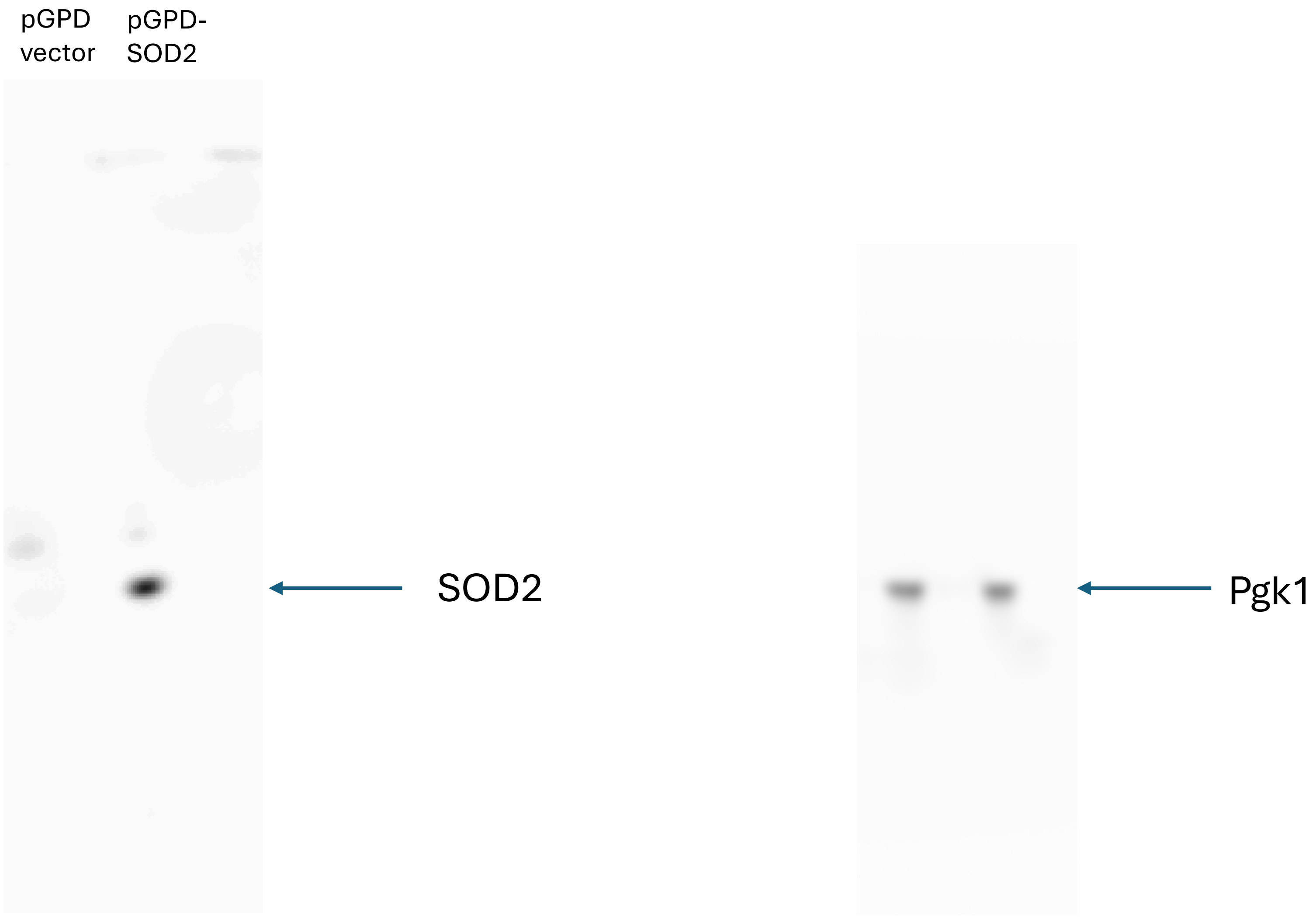

Anti Sod2

Anti Pgk1

Related to Fig 10a

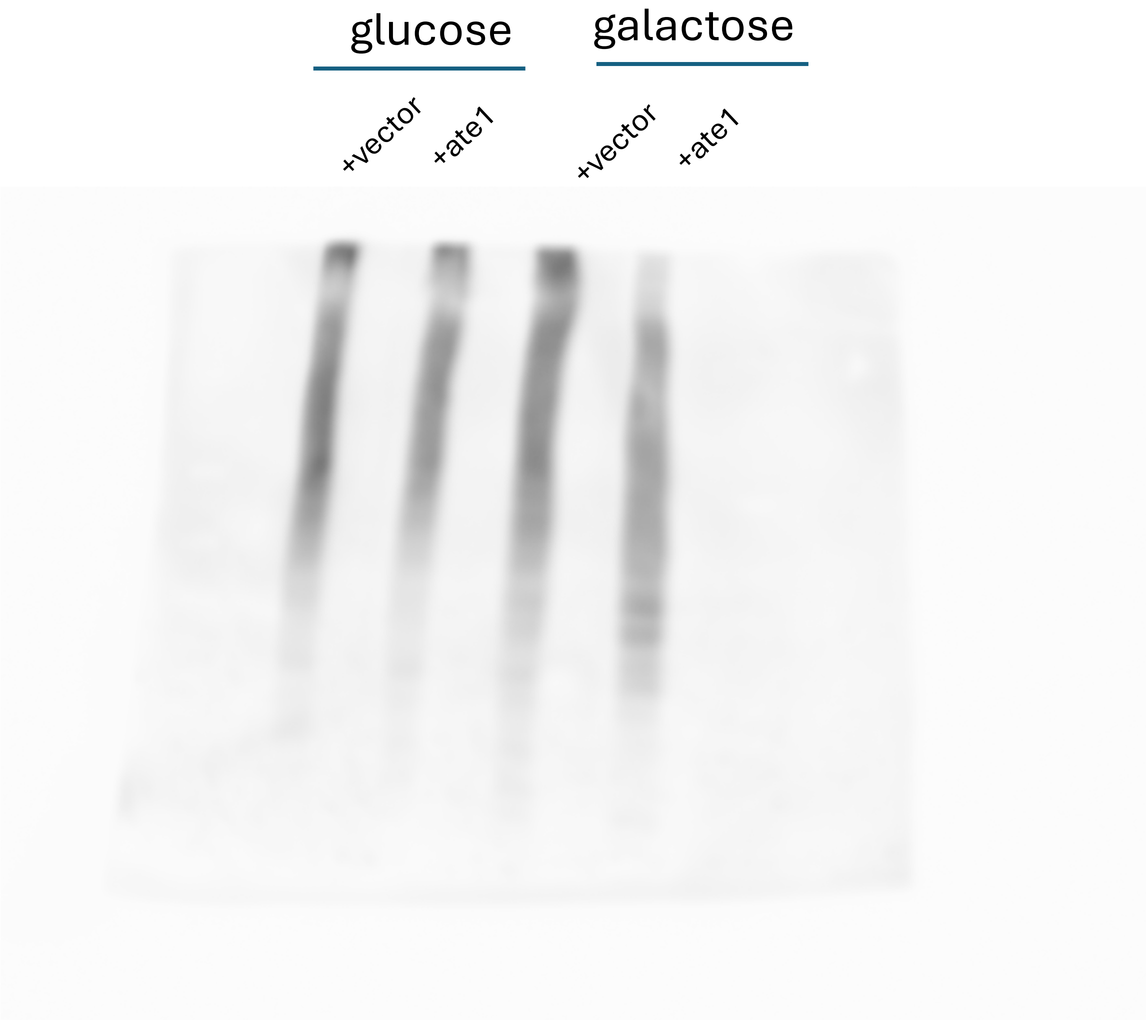

Anti ubiquitin

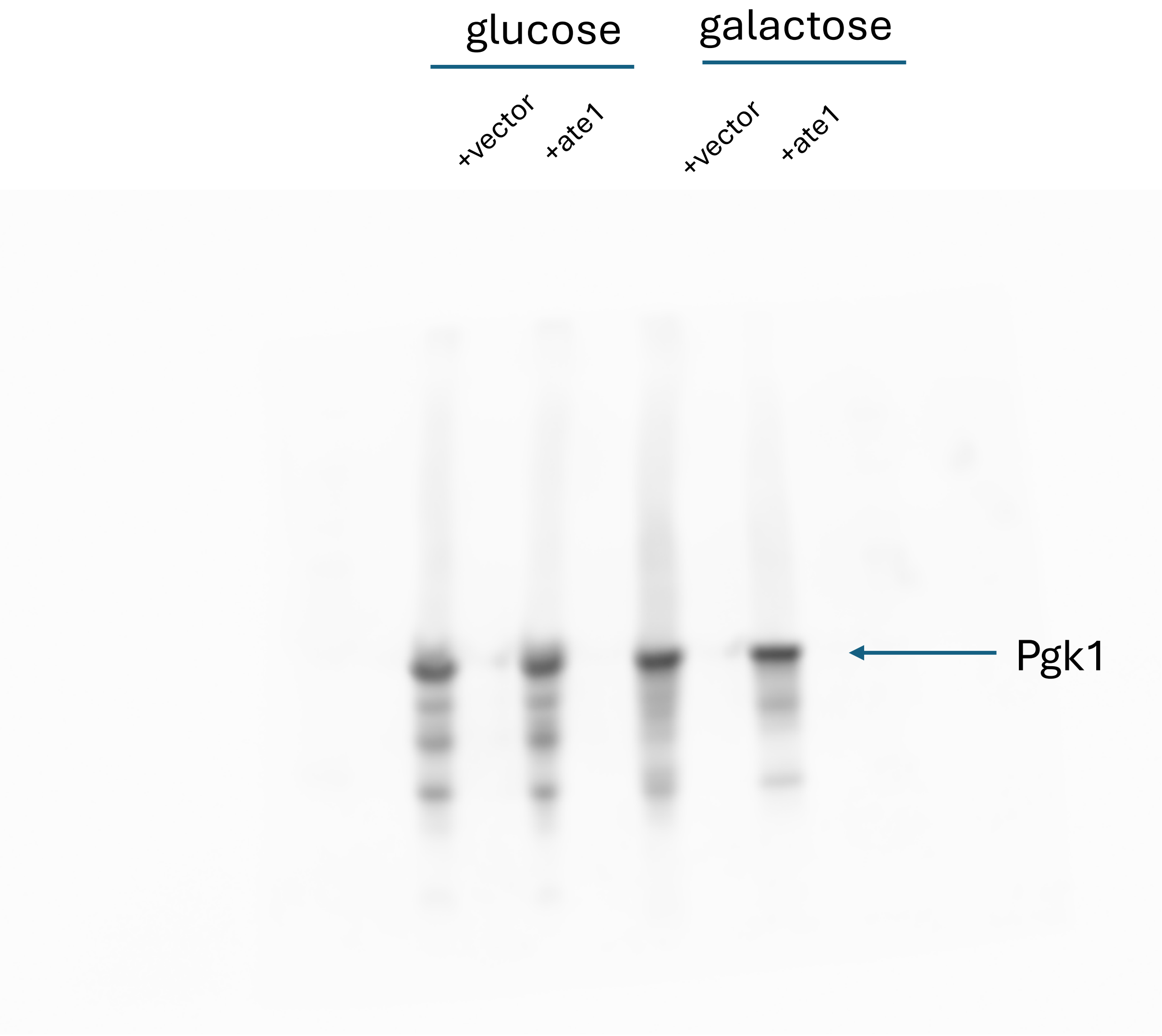

Anti-Pgk1

Related to Figure 10B

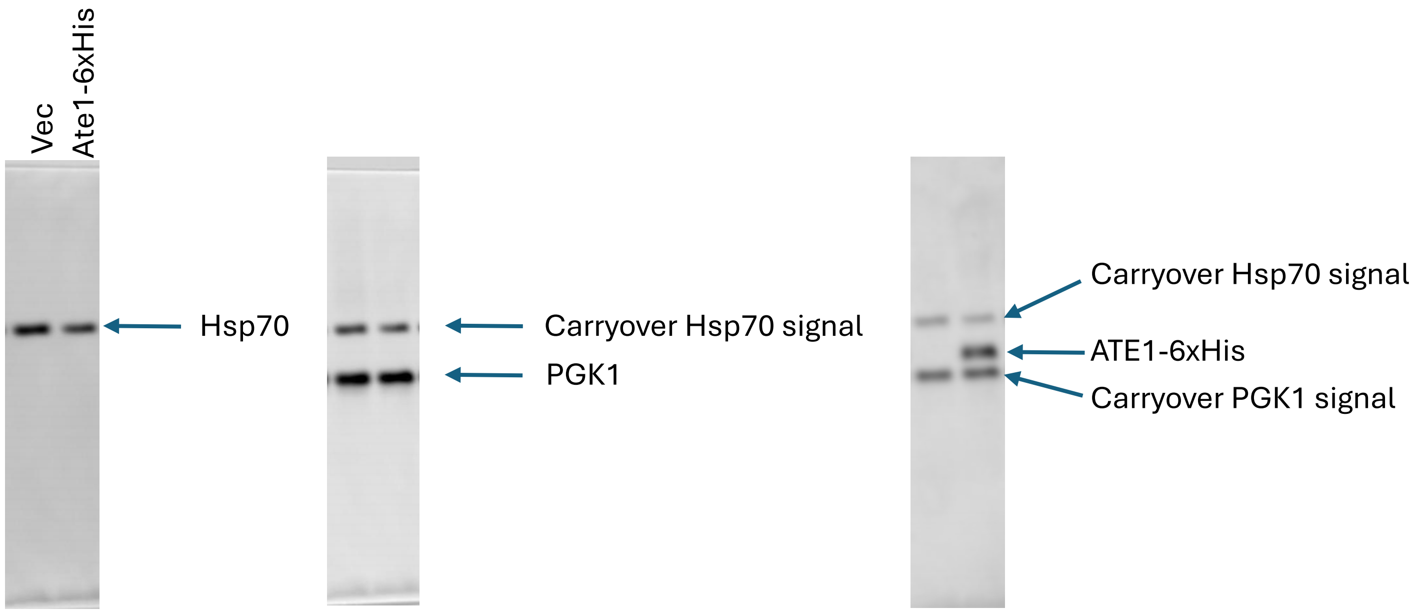

Related to Figure 10C

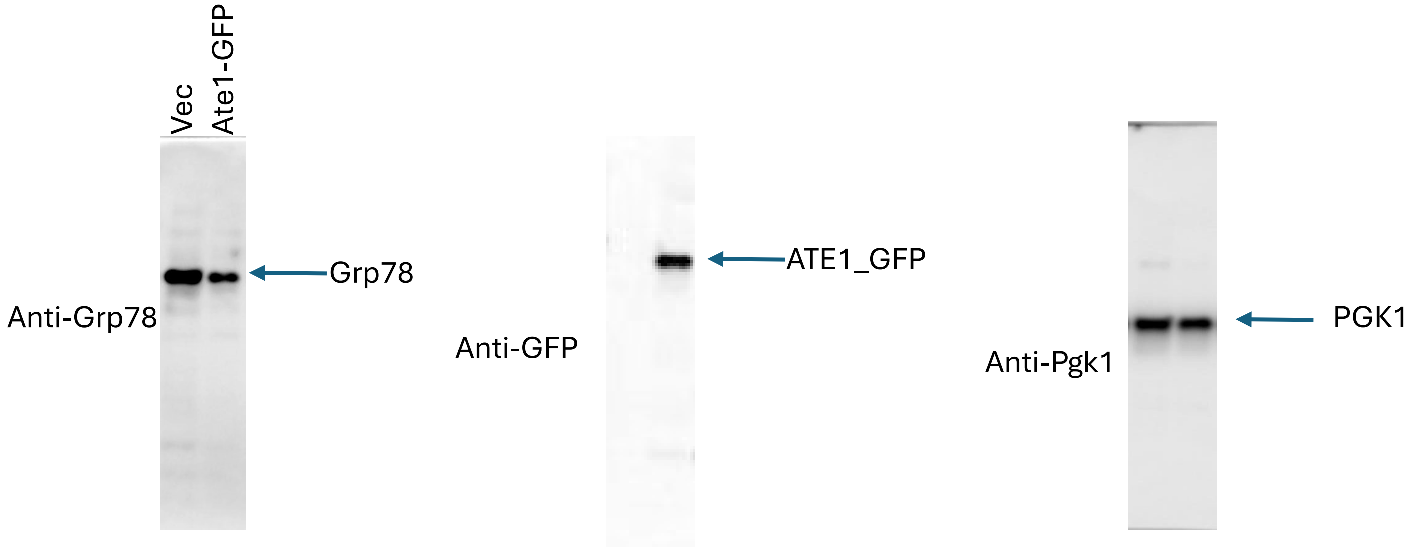

Related to Suppl Fig. S1A

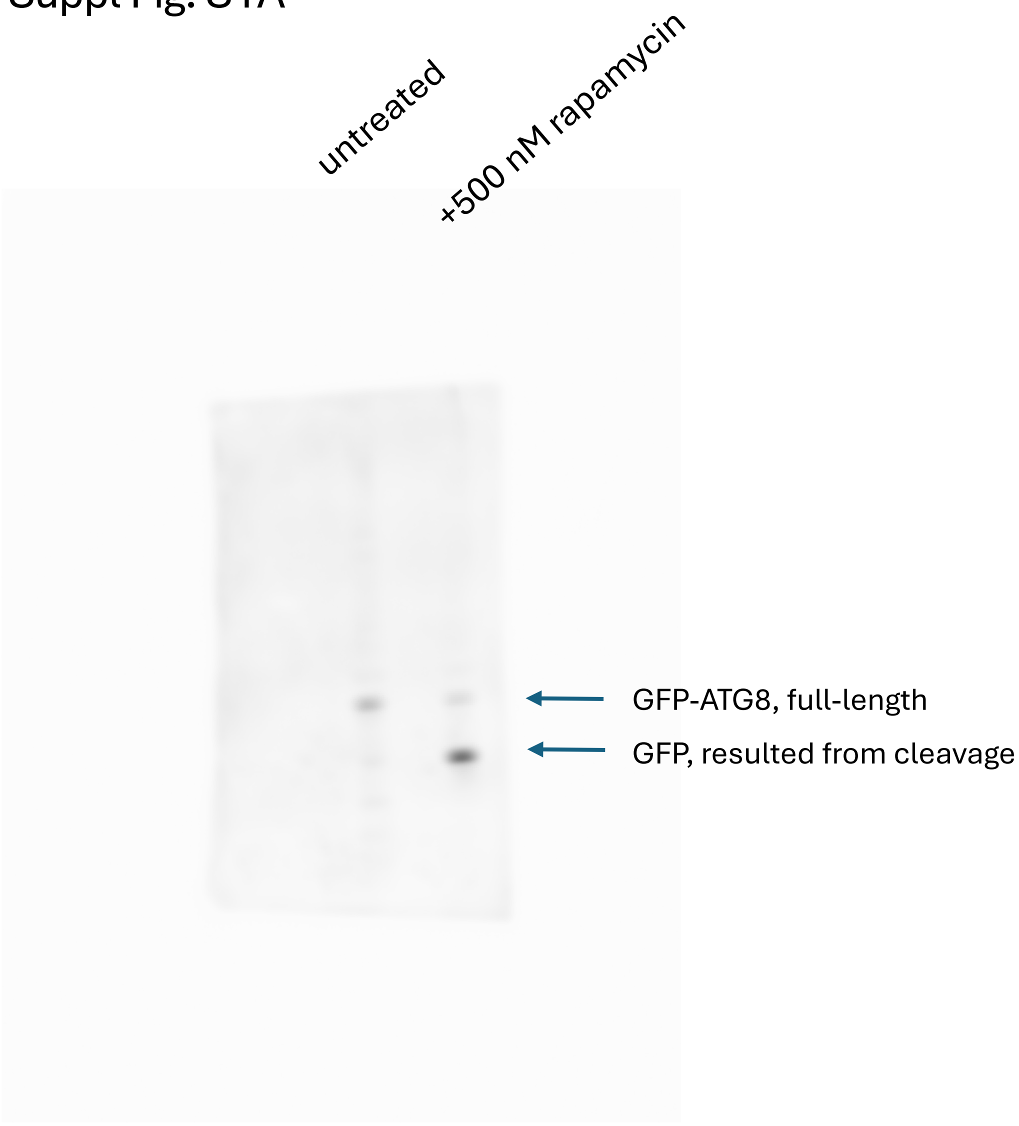

Anti-GFP

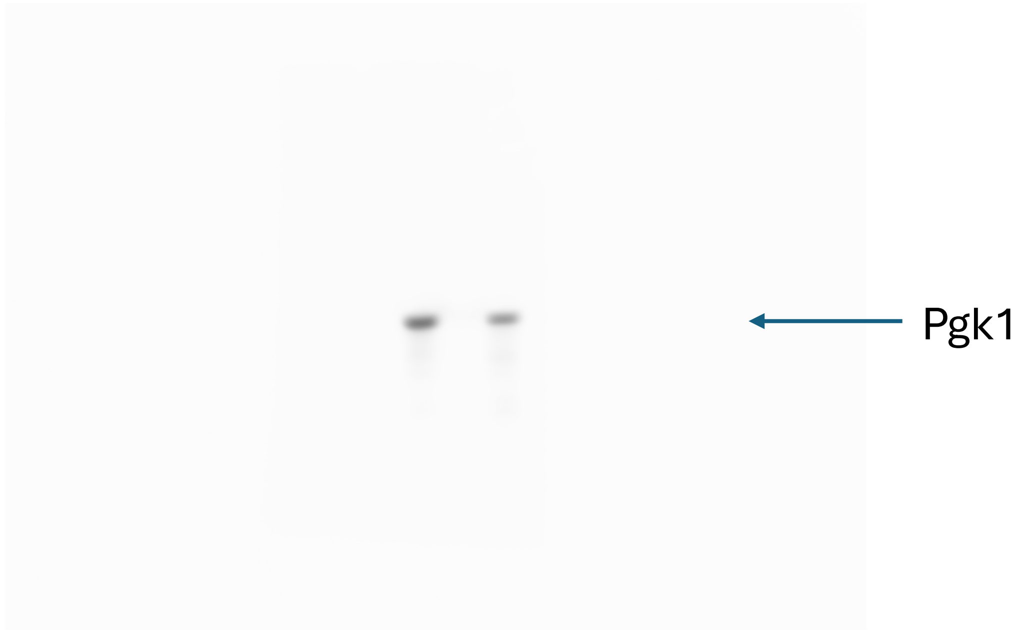

Anti-Pgk1
